# Sex differences in the microglial response to stress and chronic alcohol exposure in mice

**DOI:** 10.1186/s13293-025-00701-y

**Published:** 2025-03-04

**Authors:** Alexa R. Soares, Vernon Garcia-Rivas, Caroline Fai, Merrilee Thomas, Xiaoying Zheng, Marina R. Picciotto, Yann S. Mineur

**Affiliations:** 1 Department of Psychiatry, Yale University, 34 Park Street, 3rd Floor Research, New Haven, CT 06508 USA; 2 Yale Interdepartmental Neuroscience Program, New Haven, CT USA; 3 Department of Psychiatry, Yale University School of Medicine, 34 Park Street – 3rd Floor Research, New Haven, CT 06508 USA

**Keywords:** Microglia, Alcohol, Stress, Amygdala, Hippocampus, Immunohistochemistry, Neuroinflammation, Addiction

## Abstract

**Background:**

Women are more susceptible to stress-induced alcohol drinking, and preclinical data suggest that stress can increase alcohol intake in female rodents; however, a comprehensive understanding of the neurobiological processes underlying this sex difference is still emerging. Neuroimmune signaling, particularly by microglia, the brain’s macrophages, is known to contribute to dysregulation of limbic circuits following stress and alcohol exposure. Females exhibit heightened immune reactivity, so we set out to characterize sex differences in the microglial response to stress and alcohol exposure.

**Methods:**

Male and female C57BL/6J mice were administered alcohol over 15 or 22 trials of a modified Drinking in the Dark paradigm, with repeated exposure to inescapable footshock stress and the stress-paired context. Mice were perfused immediately after drinking and we performed immunohistochemical analyses of microglial density, morphology, and protein expression in subregions of the amygdala and hippocampus.

**Results:**

We observed dynamic sex differences in microglial phenotypes at baseline and in response to stress and alcohol. Microglia in the hippocampus displayed more prominent sex differences and heightened reactivity to stress and alcohol. Chronic alcohol exposure decreased density of amygdala microglia and lysosomal expression.

**Conclusion:**

We analyzed multiple measures of microglial activation, resulting in a comprehensive assessment of microglial changes mediated by sex, stress, and alcohol. These findings highlight the complexity of microglial contributions to the development of AUD and comorbid mood and stress disorders in men and women.

**Supplementary Information:**

The online version contains supplementary material available at 10.1186/s13293-025-00701-y.

## Highlights

At baseline, female microglia displayed reduced ramification in the amygdala and decreased soma size and lysosomal density in the hippocampus compared to males.

Alcohol altered microglial density and increased ramification in the amygdala.

Alcohol decreased expression of CD68, a lysosomal marker, in the hippocampus and amygdala.

In the hippocampus, stress had sex-specific effects on microglial morphology and protein expression.

**Supplementary Information:**

The online version contains supplementary material available at 10.1186/s13293-025-00701-y.

## Abstract (fr)

Rates of alcohol use disorder (AUD) and problematic drinking are increasing in women, and women are more likely to develop AUD in response to stressful life events. Inflammation caused by microglia, immune cells in the brain, can cause dysfunction in brain regions like the amygdala and hippocampus that mediate drinking behavior and stress reactivity. In this study, we modeled stress-induced binge drinking in male and female mice and then measured changes in microglia caused by stress and alcohol. We identified complex sex differences in the amygdala and hippocampus, and observed that microglia in the hippocampus were particularly reactive to stress and alcohol exposure. This work expands our understanding of sex differences in AUD and highlights microglia as a potential target for treatment.

## Background

In recent years, problematic drinking and alcohol use disorder (AUD) among women has increased dramatically in the United States [1]. While enrollment of women in human clinical trials has improved, studies on the brain mechanisms underlying AUD often lack female participants [2]. Notably, women are more likely to develop AUD as a result of stressful experiences [3]. The preclinical literature has also identified potentiated alcohol consumption in female rodents following stress exposure [4]. Limbic structures such as the amygdala and hippocampus (HPC) are critical for coordinating behavioral responses to stressors [5, 6]. These brain areas are also necessary for the development and persistence of addiction [7], and both regions demonstrate sex-dependent patterns of reactivity to stress [8, 9] and alcohol [10, 11].

With respect to cellular mechanisms contributing to the intersection between sex, stress and alcohol intake, neuroimmune signaling plays a key role in the dysregulation of limbic circuitry following stress [12] and alcohol exposure [13], and contributes to addiction etiology [14]. Importantly, heightened immune reactivity has consistently been observed in females [15, 16], suggesting that neuroimmune interactions may underlie female-specific vulnerabilities to stress-related drinking behavior [4]. Microglia are the brain’s resident macrophages [17]. Microglia contribute to synaptic plasticity by pruning and reshaping neuronal processes [18] and they serve as key mediators of the brain’s response to alcohol [19] and stress [20], but research on sex differences in these functions is limited [4].

Historically, microglia, like other macrophages, have been categorized into two states: M1, which predominately contribute to pro-inflammatory reactions, and M2, which primarily engage in anti-inflammatory processes [21]. More recently, debates have emerged about the appropriate classification of microglial activation states as researchers move away from this simple dichotomy, taking into account a variety of indicators of microglial function, including morphology and protein expression [17, 22, 23]. Microglial research is further complicated by the heterogeneity of functions and phenotypes across brain regions [24, 25]. More precise methods of characterizing microglia are needed to capture the nuanced variability of these cells and their function in healthy and pathological brains, and to determine how sex differences affect specific aspects of microglial structure and function.

Recent findings in the preclinical literature have demonstrated sex differences in microglial responses to stress and alcohol in limbic circuits, with mixed results. Studies in Sprague-Dawley rats [26] and C57BL/6 mice [27] have found that males exhibited more morphological reactivity to stress in the basolateral amygdala (BLA) and HPC, but others using Wistar Han rats have seen more prominent morphological changes in females [28]. Alcohol exposure has been shown to increase HPC microglial density in females [29], although this effect may be subfield-specific [30]. Of course, differences in stress and alcohol administration paradigms also contribute to variation in these patterns.

Given the importance of microglial function in stress and alcohol addiction, and the need to understand AUD pathology in women [31], we investigated sex differences in microglial reactivity to stress and alcohol exposure in a mouse model of stress-induced binge drinking. We hypothesized that microglial phenotypes might be altered synergistically in limbic structures by exposure to stress and alcohol, and recruited differentially in male and female mice. Through a comprehensive immunohistochemical analysis of cell density, morphology, and protein expression, we characterized sex- and region-specific effects of stress and alcohol on microglial phenotypes in the amygdala and HPC.

## Methods

### Animal husbandry

Male and female (defined by large vs. small anogenital distance at weaning) C57BL/6J mice of 8–10 weeks of age (The Jackson Laboratory) were maintained on a reverse 12-hr light/dark cycle (lights off at 11:00 AM) with *ad libitum* access to food and water. For one week of acclimation, mice were group-housed and handled daily, before switching to single-housing for the remainder of the experiment. All procedures were approved by the Yale University Institutional Animal Care and Use Committee and were carried out in compliance with the National Institute of Health’s Guide for the Care and Use of Laboratory Animals.

### EtOH exposure

EtOH solutions were prepared fresh by diluting undenatured 100% EtOH (Decon Laboratories) in filtered drinking water. To acclimate mice to the taste and pharmacological effects of EtOH before two-bottle volitional choice of EtOH vs. water, mice were exposed to increasing concentrations of EtOH diluted in water (5% EtOH v/v for 3 days, 10% EtOH v/v for 4 days) as their sole liquid source for one week. On the last day of passive EtOH exposure, bottles were replaced with regular water bottles prior to lights off. Mice drank EtOH in their home cage through a limited access paradigm (Drinking-in-the-Dark: DiD), based on Rhodes et al. [32]. Access to EtOH was limited to 5 days a week. 3 h after lights off, water bottles were removed and two sippers, one containing water and one containing 10% EtOH, were introduced into the home cage for 2 h. On selected days, access to EtOH was extended to 4 h to mimic an escalated binge.

### Stress exposure

On stress exposure days, *Stress* mice were placed individually into a chamber containing an electrified floor grate (Med Associates, VT) inside a sound-attenuating box. Mice received 120 low-intensity inescapable footshocks (0.3 mA, 4 s), delivered at semi-random intervals (1–17 s) over the course of 1 h [33–36]. On re-exposure days, *Stress* mice were placed into the stress-associated chamber for 5 min without footshocks. Stress and re-exposure trials occurred at least 4 h prior to DiD, at the end of the light phase, when mice are usually less active, so that the footshock stress could potentially be compounded by disruption of the circadian rhythm. *No Stress* mice remained in their home cages undisturbed.

### Experimental design

*EtOH* mice (*n* = 24 animals/sex) underwent passive EtOH exposure followed by 11 DiD trials (T), as described above. At the end of this phase (T11), mice were randomized into *Stress* and *No Stress* groups (*n* = 12 animals/sex/group). *Stress* mice experienced the stress exposure paradigm, as described above, prior to T12 and T13, and re-exposure prior to T15. A subset of mice (*n* = 6 animals/sex/group) was perfused following DiD on T15 and brains were collected for immunohistochemistry (IHC). The remaining mice (*n* = 6 animals/sex/group) continued for 7 additional DiD trials, with re-exposure prior to T17 and T19, and stress exposure on T21 and T22. These mice were perfused for IHC following DiD on T22. Control *No EtOH* animals were age-matched, single-housed male and female mice housed in the same facility but not exposed to EtOH. *No EtOH* mice were also randomized into *Stress* and *No Stress* groups (*n* = 12 animals/sex/group), subjected to the same stress and re-exposure paradigms described above, and perfused for IHC on T15 and T22 (*n* = 6 animals/sex/group/trial) alongside *EtOH* mice. See Fig. . 1A for timeline.

### Immunohistochemistry

Mice were anesthetized with pentobarbital (Fatal-Plus, Vortech Pharmaceuticals) and intracardially perfused with ~ 50 mL of 4% paraformaldehyde (PFA; Electron Microscopy Sciences) in phosphate-buffered saline (PBS; Gibco) immediately after the final DiD trial. Brains were extracted and post-fixed for 1 day in 4% PFA at 4 °C before transferring to 30% sucrose (Millipore Sigma) in PBS at 4 °C. Brains were sectioned on a freezing microtome (Leica) at 40 μm and slices were stored in 0.02% sodium azide (Millipore Sigma) PBS solution at 4 °C. For IHC analyses, sections were washed in PBS and incubated in a 0.3% Triton-X (American Bioanalytical) PBS solution for 15 min at room temperature (RT). Sections were then rinsed in PBS for 5 min and incubated in 0.01 M citric acid (Millipore Sigma) for 30 min at 70 °C for antigen retrieval, followed by PBS rinsing for 5 min, and then 1 h in a sodium tetraborate (Millipore Sigma) buffer at RT. After subsequent PBS rinsing for 5 min, sections were blocked in 3% normal donkey serum (NDS; Jackson Immuno) and 0.3% Triton-X in PBS for 1 h at RT. Sections were then incubated in primary antibodies (Table 1) and PBS overnight at 4 °C. On day 2, sections were rinsed in PBS for 5 min and incubated in secondary antibodies (Table 1) in PBS for 1 h at RT. Sections were rinsed in PBS for 5 min, then mounted on slides and coverslipped with ProLong Gold antifade mounting medium (Invitrogen).

**Table 1:** Antibody information for IHC experiments

Analysis	[Concentration] Primary Antibody (Source)	[Concentration] Secondary Antibody (Source)
Morphology/Lysosome	[1:1000] rabbit anti-P2Y12 (AnaSpec AS-55043A)	[1:2000] donkey anti-rabbit Alexa 647 (Invitrogen A-31573)
Morphology/Lysosome	[1:1000] goat anti-Iba1 (WAKO 011-27991)	[1:1000] donkey anti-goat Alexa 488 (Invitrogen A-11055)
Morphology/Lysosome	[1:1000] rat anti-CD68 (BioRad MCA1957T)	[1:1000] donkey anti-rat Alexa 555 (Abcam ab150154)
Polarization	[1:1000] rabbit anti-iNos (Abcam ab15323)	[1:2000] donkey anti-rabbit Alexa 555 (Invitrogen A-31572)
Polarization	[1:1000] goat anti-Iba1 (WAKO 011-27991)	[1:1000] donkey anti-goat Alexa 647 (Invitrogen A32849)
Polarization	[1:500] chicken anti-Arg1 (Sigma-Aldrich ABS535)	[1:1000] donkey anti-chicken Alexa 488 (Sigma-Aldrich SAB4600031)

### Confocal microscopy

Z-stack images were acquired in 5 serial sections at 60x magnification with a 1.0 μm increment between slices using a FLUOVIEW FB10i confocal microscope (Olympus). Acquired images were analyzed in FIJI [37] and Matlab R2022a (MathWorks) using custom codes to measure microglial density, morphology, lysosomal density, and polarization state, as described below.

### Morphology analysis

**Fig. 1:**
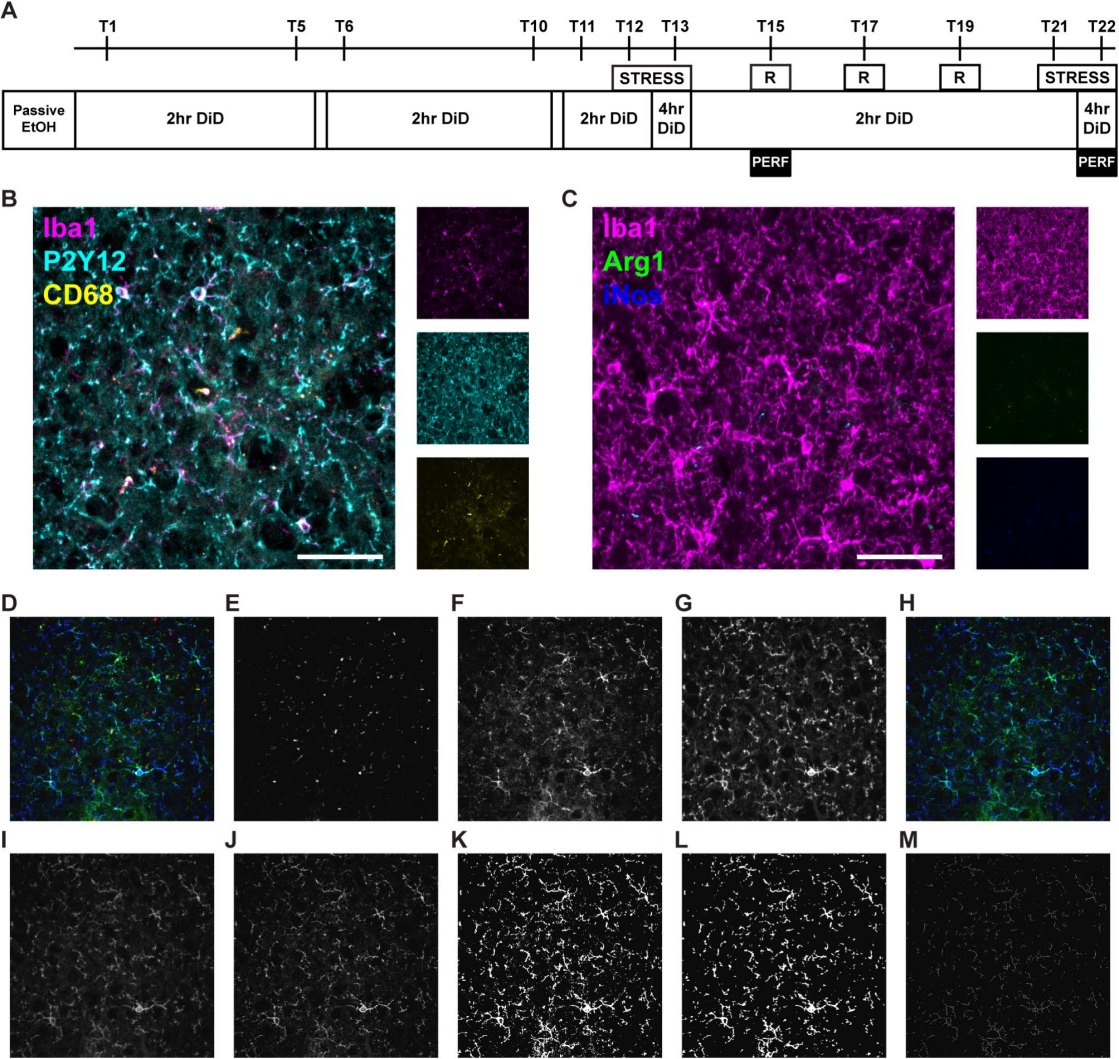
Experimental design. **(A)** Alcohol and stress exposure timeline. EtOH = ethanol; DiD = drinking in the dark; R = re-exposure; T = trial; PERF = perfusion. **(B)** Example micrograph for the Morphology/Lysosome stain, with individual channels to the right. Magenta = Iba1; cyan = P2Y12; yellow = CD68; scale bar = 50 μm. **(C)** Example micrographs for the Polarization stain, with individual channels to the right; Magenta = Iba1; green = Arg1; blue = iNos; scale bar = 50 μm. Example micrographs for the Morphology image analysis pipeline: **(D)** Original multi-channel image. **(E)** Red channel from original image **(D)**. **(F)** Green channel from original image **(D)**. **(G)** Blue channel from original image **(D)**. **(H)** Green (F) and blue (G) channels merged. **(I)** Merged image **(H)** converted to grayscale. **(J)** Grayscale image **(I)** after application of Unsharp Mask and Despeckle tools. **K.** De-noised image (J) after thresholding. **L.** Thresholded image (K) after application of Despeckle, Close-, and Remove Outliers tools. **M.** Final skeletonized image used to quantify branching. For printing purposes, all images are shown as Max Intensity Z-Projections

Multichannel Z-stack images were acquired in 5 serial sections at 60x magnification using a FLUOVIEW FV10i confocal microscope (Olympus). Regions of interest were outlined using pre-defined boundaries and landmarks in accordance with a mouse brain atlas [38]. 2–5 images each were captured within the BLA and CeA between bregma − 0.59 mm and bregma − 1.55 mm. 2–3 images were captured for each HPC subfield between bregma − 1.43 mm and bregma − 2.15 mm. Acquired images (see Fig. 1D for a representative micrograph) were analyzed first in FIJI [37] using a custom Macro code based on Young & Morrison [39] and subsequently in Matlab R2022a (MathWorks) using custom code. Green (Iba1; Fig. 1F) and blue (P2Y12; Fig. 1G) channels were merged to form a single representation of microglia. A Z-Projection of this Composite stack was created based on “Max Intensity” (Fig. 1H) and converted to Grayscale (Fig. 1I). “Unsharp Mask” and “Despeckle” tools were used to sharpen edges and remove noise (Fig. 1J). The number of somas in each Composite stack was counted manually using the “Multipoint” tool. Microglial density was determined by summing this count across each image for a given region of interest and dividing by the total area imaged for that region in each animal. Soma size was determined by tracing each soma using the “Freehand” tool and calculating the area using the “Measure” tool, then dividing by the total number of somas for each region of interest in each animal. Thresholding (Fig. 1K) and the “Despeckle”, “Close-“, and “Remove Outliers” tools (Fig. 1L) were applied to the de-noised Composite Z-projection, which was then further processed using the “Skeletonize” tool (Fig. 1M). The “Skeleton” plugin was used to quantify branch length and branch number, which were both normalized to the total number of somas.

### Lysosome analysis

The same raw Z-stacks acquired for the Morphology analysis were used for the Lysosome analysis. Acquired images were analyzed first in FIJI [37] using a custom Macro code and subsequently in Matlab R2022a (MathWorks) using custom code. Green (Iba1; Fig. 1F) and blue (P2Y12; Fig. 1G) channels were merged to form a single representation of the microglia (Fig. 1H). The Composite stack was converted to 8-bit and then background was corrected across the stack using the “Threshold” tool before binarizing and Despeckling to remove noise. The “Analyze Particles” tool was used to quantify the total area covered by microglia as represented by expression of Iba1 and/or P2Y12, and this number was summed across all the images for a given region of interest for each animal. The stack for the red channel (Fig. 1E) was similarly Thresholded, binarized, and Despeckled. The “Image Calculator” tool was used to calculate colocalization between the red stack and the Composite stack, and this was summed across images and normalized to the total Iba1/P2Y12^+^ area to determine the percentage of CD68 expression in microglia.

### Polarization analysis

Multichannel Z-stacks were acquired as described above. Acquired images were analyzed first in FIJI [37] using a custom Macro code and subsequently in Matlab R2022a (MathWorks) using custom code. Channels were split. The blue (Iba1) channel was duplicated and a Z-Projection was created using “Max Intensity” to measure microglial density as described above. To measure colocalization, the original blue stack was used. Background was corrected across the stack using the “Threshold” tool before binarizing and Despeckling to remove noise. The “Analyze Particles” tool was used to measure the Iba1^+^ area. The “Image Calculator” tool was used to calculate colocalization between the red (iNos) and blue channels, and this was summed across all of the images for a given region of interest and normalized to the total Iba1^+^ area to determine percentage of iNos expression in microglia for each animal. These steps were repeated using the green (Arg1) and blue channels to determine the percentage of Arg1 expression in microglia. The percentage of iNos colocalization was divided by the percentage of Arg1 colocalization to determine the ratio of iNos: Arg1 expression in microglia.

### Statistical analyses

Statistical analyses were performed in GraphPad Prism 10 and Microsoft Excel 2021. Results are presented as the mean ± standard error of the mean (SEM). 3-way ANOVAs were performed with Sex, Stress, and EtOH as between-subject factors. Significant interactions with Sex were followed up with 2-way ANOVAs within Sex (Stress x EtOH) and *post-hoc* Šídák’s multiple comparisons t-tests when appropriate. All analyses are available in supplementary tables, with only the significant effects (*p* < 0.05) reported in the main text. Effect sizes are presented as partial eta squared (*η*_*p*_^2^) for ANOVAs and Cohen’s d (*d*) for t-tests. Outliers were determined using Grubbs’ test (α < 0.05) and subsequently excluded.

## Results

The goal of these experiments was to assess comprehensively how sex, stress exposure, and EtOH drinking affect microglial number and morphology over time. The first cohort of mice underwent the full EtOH/stress exposure paradigm, consisting of 3 weeks of DiD, the first stress induction, 3 re-exposures to the stress context, and a second stress induction ending on T22. We noticed an early escalation of EtOH drinking behavior following the first re-exposure [40], so we analyzed a second cohort of mice that underwent an abbreviated paradigm ending with the first re-exposure on T15. To determine how microglial changes evolve over this paradigm, we performed 2 triple-stain IHC analyses to measure different aspects of microglial morphology and protein expression on T15 and T22 (Fig. 1A). The first stain (Fig. 1B) combined expression of Iba1, a widely-used microglial marker [41], and P2Y12, which is enriched in microglial processes [42, 43], to generate a more complete image of the microglial cells and their branches. This allowed us to measure density, soma size, branch number, and branch length, components of cell morphology that are tied to microglial reactivity [44]. Smaller somas with increased branching represent a ramified state; as microglia become stimulated, they shift towards an ameboid phenotype, with larger somas and decreased branching [45]. We also measured colocalization of CD68 with Iba1 and P2Y12; CD68 is a marker of microglial lysosomes that is upregulated in actively phagocytic cells, so increased staining may indicate increased clearance of debris and synapses [46–48]. In the second stain (Fig. 1C), we compared expression of iNos, a traditional M1 marker, and Arg1, an M2 marker, in Iba1^+^ microglia [49]. Although the M1/M2 dichotomy was established for the categorization of peripheral macrophages and may not map on well to microglia [23], these two markers have been shown to differentiate between classically activated microglia that release pro-inflammatory cytokines and reactive oxygen species, and alternatively activated microglia that release anti-inflammatory cytokines and drive tissue repair functions in rodent models of in vivo immune challenges [49, 50]. iNos uses arginine to synthesize nitric oxide, while Arg1 competes with iNos, using arginine for wound healing; comparing relative levels of these enzymes provides insight into whether arginine is being used by microglia to produce reactive oxygen species or to downregulate inflammation [49]. Together, these measurements of morphology and protein expression allow for assessment of microglial phenotypes that goes beyond dichotomous categorization schemes (17,22).

### Microglia are more reactive to EtOH in the male BLA

**Fig. 2:**
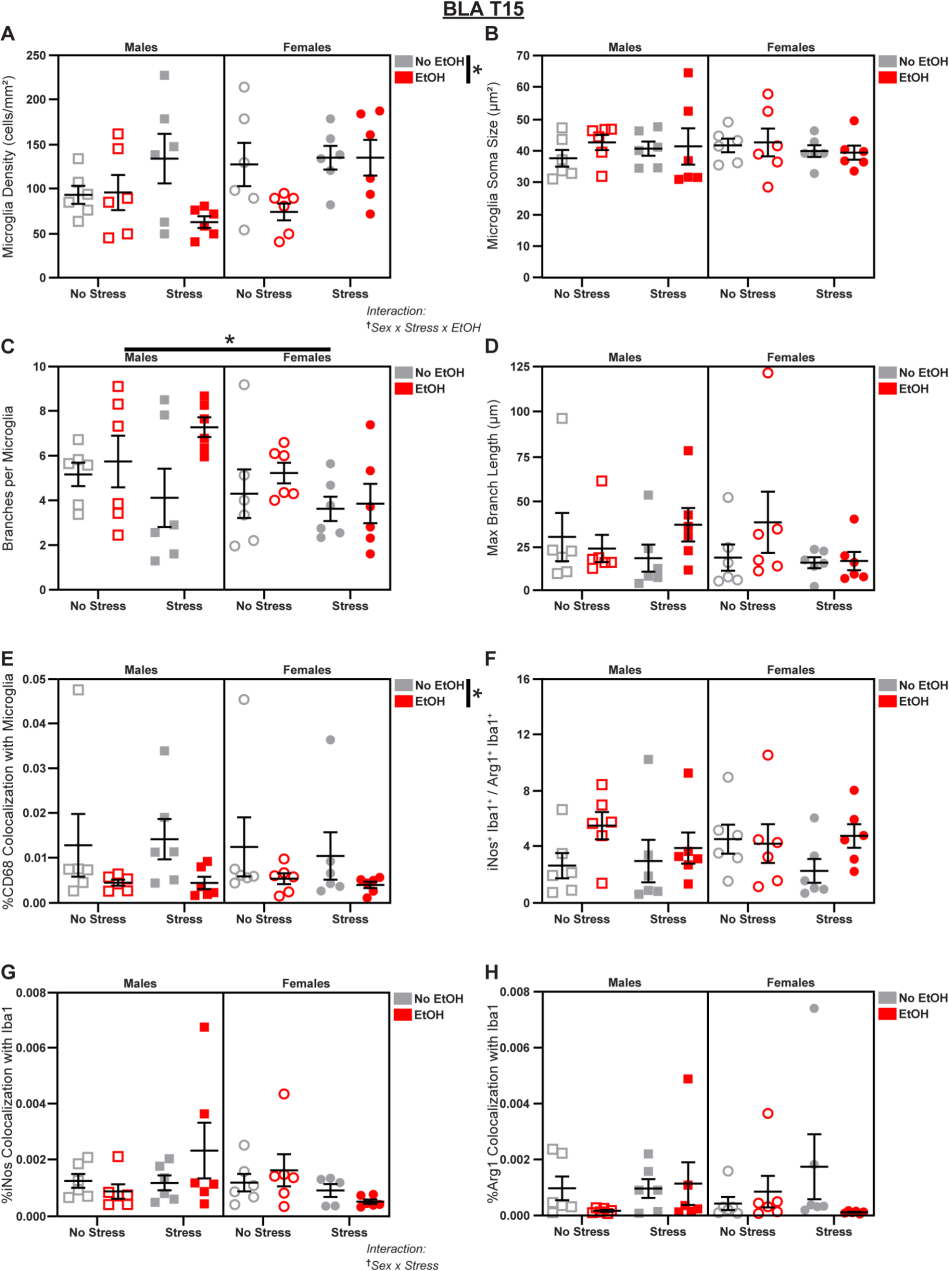
Microglial changes in the BLA at T15. Effects of sex, stress, and EtOH on **(A)** microglial density, **(B)** soma size, **(C)** branch number, **(D)** branch length, **(E)** CD68 expression (2 outliers excluded), **(F)** the iNos: Arg1 ratio, **(G)** iNos expression (1 outlier excluded), and **(H)** Arg1 expression. Lines outside of graphs indicate a main effect of sex or EtOH in 3-way ANOVAs, *p < 0.05. Text outside of graphs indicates two- or three-way interactions in 3-way ANOVAs, ^†^*p* < 0.05. *n* = 5–6 animals/group

Within the amygdala, we first examined the BLA, a structure exhibiting profound sex differences in molecular and cellular architecture, as well as circuit-level connectivity, that plays a key role in the etiology of AUD and stress disorders [51]. On T15, we observed several changes induced by EtOH exposure. EtOH decreased microglial density (main effect of EtOH in 3-way ANOVA: *F*_1,40_ = 5.776; *p* = 0.0210; *η*_*p*_^2^ = 0.1262; Fig. 2A), but stress reversed the EtOH-induced decrease in females (Sex x Stress x EtOH interaction in 3-way ANOVA: *F*_1,40_ = 6.728; *p* = 0.0164; *η*_*p*_^2^ = 0.1357; Fig. 2A; see Table S1 for follow-ups). EtOH also decreased microglial CD68 expression (main effect of EtOH in 3-way ANOVA: *F*_1,40_ = 6.936; *p* = 0.0120; *η*_*p*_^2^ = 0.1478; Fig. 2E).

We observed a Sex x Stress interaction (3-way ANOVA: *F*_1,39_ = 4.455; *p* = 0.0413; *η*_*p*_^2^ = 0.1025; Fig. 2G; see Table S1 for follow-ups) in microglial iNos expression that may suggest a stress-induced decrease in iNos among females. However, this did not correspond to a change in the iNos: Arg1 ratio (Fig. 2F), or any effects on microglial Arg1 expression (Fig. 2H). Finally, we observed a baseline sex difference, with males displaying increased branch number compared to females (main effect of Sex in 3-way ANOVA: *F*_1,40_ = 4.683; *p* = 0.0365; *η*_*p*_^2^ = 0.1048; Fig. 2C). There were no significant effects of sex, stress, or EtOH on soma size (Fig. 2B) or branch length (Fig. 2D) at T15; see Fig. S1 for representative micrographs and Table S1 for complete statistical analyses.

**Fig. 3:**
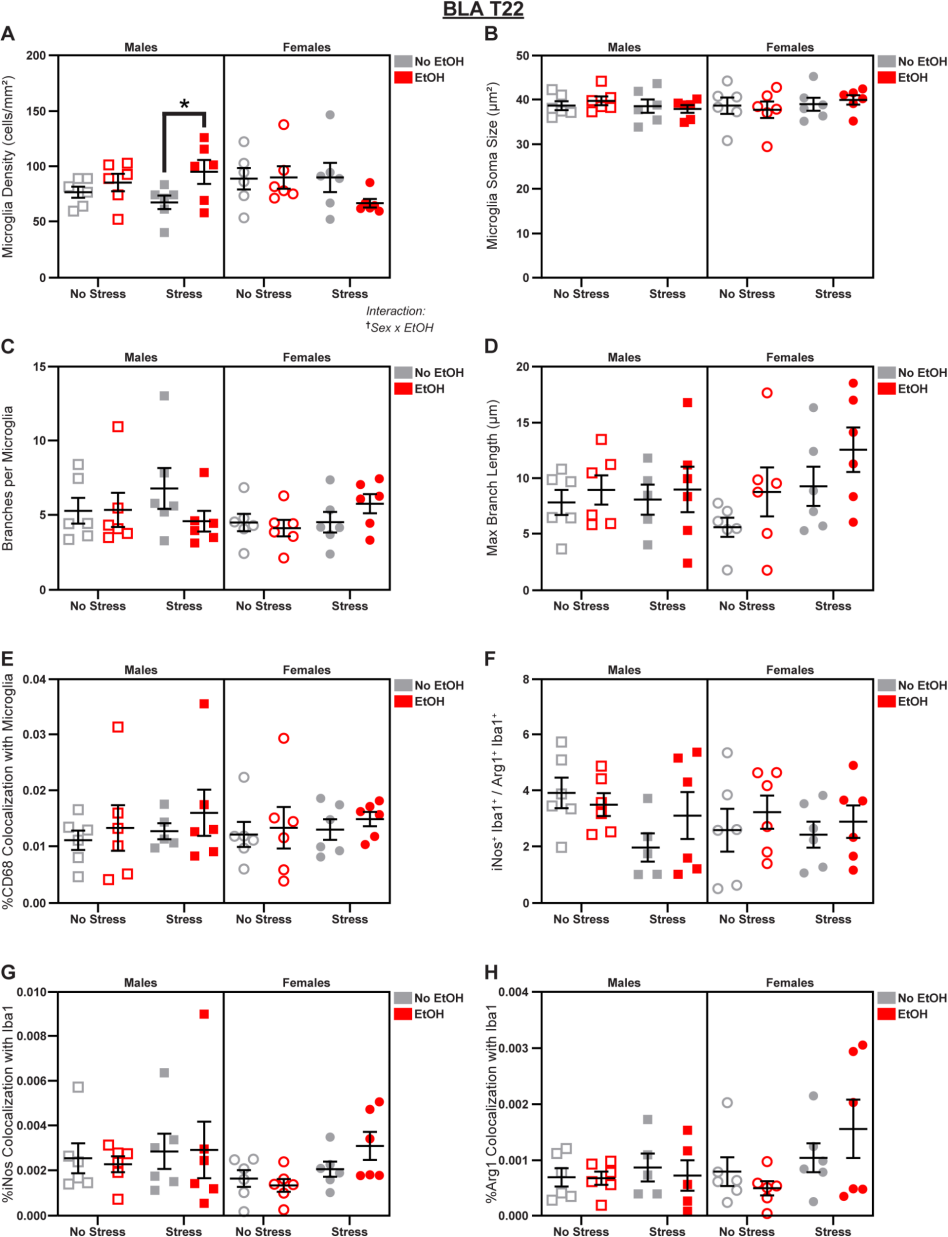
Microglial changes in the BLA at T22. Effects of sex, stress, and EtOH on **(A)** microglial density (1 outlier excluded), **(B)** soma size, **(C)** branch number, **(D)** branch length, **(E)** CD68 expression (1 outlier excluded), **(F)** the iNos: Arg1 ratio (1 outlier excluded), **(G)** iNos expression, and **(H)** Arg1 expression (2 outliers excluded). Text outside of graphs indicates two-way interactions in 3-way ANOVAs, ^†^*p* < 0.05. Lines within graphs indicate group differences in *post-hoc* Sidak’s multiple comparisons tests, *p < 0.05. *n* = 5–6 animals/group

At T22, we observed a Sex x EtOH interaction (3-way ANOVA: *F*_1,40_ = 5.425; *p* = 0.0250; *η*_*p*_^2^ = 0.1194; Fig. 3A) on microglia density; *post-hoc* analyses revealed an EtOH-induced increase in density specific to males (main effect of EtOH in Male 2-way ANOVA: *F*_1,20_ = 5.443; *p* = 0.0302; *η*_*p*_^2^ = 0.1866; Fig. 3A), driven by the *Stress* group (Šídák’s t-test, Males Stress/No EtOH vs. Stress/EtOH: *t* = 2.507; *p* = 0.0414; *d* = 1.447; Fig. 3A). There were no significant effects of sex, stress, or EtOH on soma size (Fig. 3B), branch number (Fig. 3C), branch length (Fig. 3D), CD68 expression (Fig. 3E), the iNos: Arg1 ratio (Fig. 3F), iNos expression (Fig. 3G), or Arg1 expression (Fig. 3H); see Fig. S1 for representative micrographs and Table S1 for complete statistical analyses.

Together, these data suggest that chronic EtOH administration had early effects on BLA microglia, reducing density and CD68 expression, particularly among males, but these effects were not present after an additional week of drinking. In fact, at T22, EtOH had the opposite effect and actually increased microglial density in males, especially those subjected to additional stress. Furthermore, stress had sex-specific effects on microglial expression of the oxidative stress marker iNos.

### CeA microglia display dynamic sex differences

**Fig. 4:**
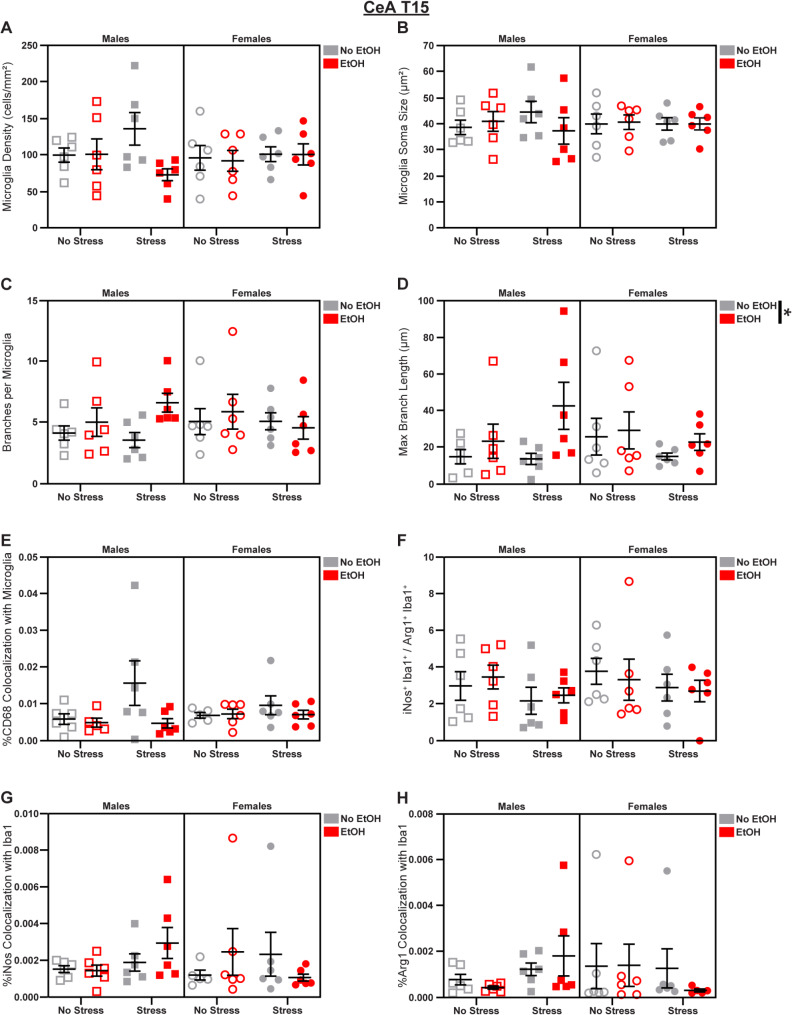
Microglial changes in the CeA at T15. Effects of sex, stress, and EtOH on **(A)** microglial density, **(B)** soma size, **(C)** branch number, **(D)** branch length, **(E)** CD68 expression (2 outliers excluded), **(F)** the iNos: Arg1 ratio, **(G)** iNos expression (1 outlier excluded), and **(H)** Arg1 expression (1 outlier excluded). Lines outside of graphs indicate main effects of EtOH in 3-way ANOVAs, *p < 0.05. *n* = 4–6 animals/group

The BLA projects heavily to the central nucleus of the amygdala (CeA), another area that mediates interactions between stress and alcohol [52], so we next characterized microglial phenotypes in the CeA. On T15, we saw that EtOH increased microglial branch length (main effect of EtOH in 3-way ANOVA: *F*_1,40_ = 4.727; *p* = 0.0357; *η*_*p*_^2^ = 0.1057; Fig. 4D). There were no significant effects of sex, stress, or EtOH on microglial density (Fig. 4A), soma size (Fig. 4B), branch number (Fig. 4C), CD68 expression (Fig. 4E), the iNos: Arg1 ratio (Fig. 4F), iNos expression (Fig. 4G), or Arg1 expression (Fig. 4H) at T15; see Fig. S2 for representative micrographs and Table S2 for complete statistical analyses.

**Fig. 5:**
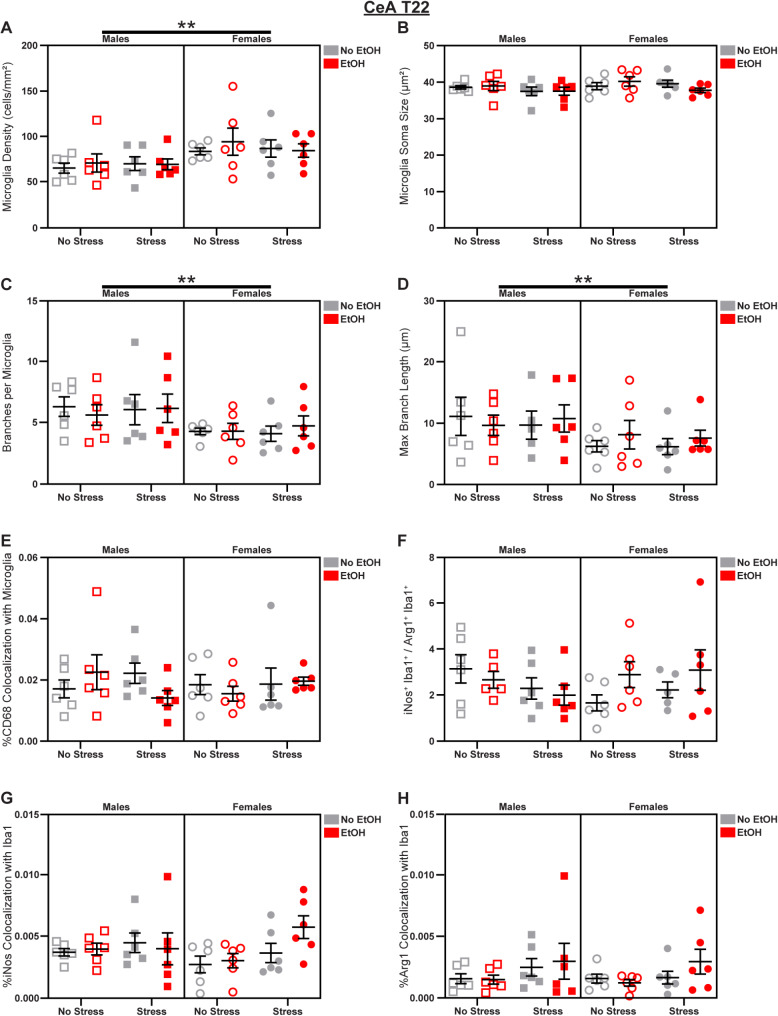
Microglial changes in the CeA at T22. Effects of sex, stress, and EtOH on **(A)** microglial density, **(B)** soma size (1 outlier excluded), **(C)** branch number, **(D)** branch length (1 outlier excluded), **(E)** CD68 expression, **(F)** the iNos: Arg1 ratio (2 outliers excluded), **(G)** iNos expression, and **(H)** Arg1 expression. Lines outside of graphs indicate main effects of sex in 3-way ANOVAs, **p < 0.01. *n* = 4–6 animals/group

Sex differences emerged in the CeA at T22, highlighting the dynamism of microglial morphology [53]. Females displayed increased microglial density (main effect of Sex in 3-way ANOVA: *F*_1,40_ = 8.986; *p* = 0.0047; *η*_*p*_^2^ = 0.1834; Fig. 5A) and reductions in branch number (main effect of Sex in 3-way ANOVA: *F*_1,40_ = 7.910; *p* = 0.0076; *η*_*p*_^2^ = 0.1651; Fig. 5C) and branch length (main effect of Sex in 3-way ANOVA: *F*_1,39_ = 0.5371; *p* = 0.0258; *η*_*p*_^2^ = 0.1211; Fig. 5D). There were no effects of sex, stress, or EtOH on soma size (Fig. 5B), CD68 expression (Fig. 5E), the iNos: Arg1 ratio (Fig. 5F), iNos expression (Fig. 5G), or Arg1 expression (Fig. 5H); see Fig. S2 for representative micrographs and Table S2 for complete statistical analyses.

These analyses revealed increased ramification of CeA microglia as a result of EtOH administration earlier in the paradigm. At T22, EtOH and stress did not significantly alter microglial phenotypes, but sex differences emerged suggesting more dense microglia, with less branching, in females across experimental groups.

### In CA1, stress and EtOH have opposite effects in male vs. female mice

**Fig. 6:**
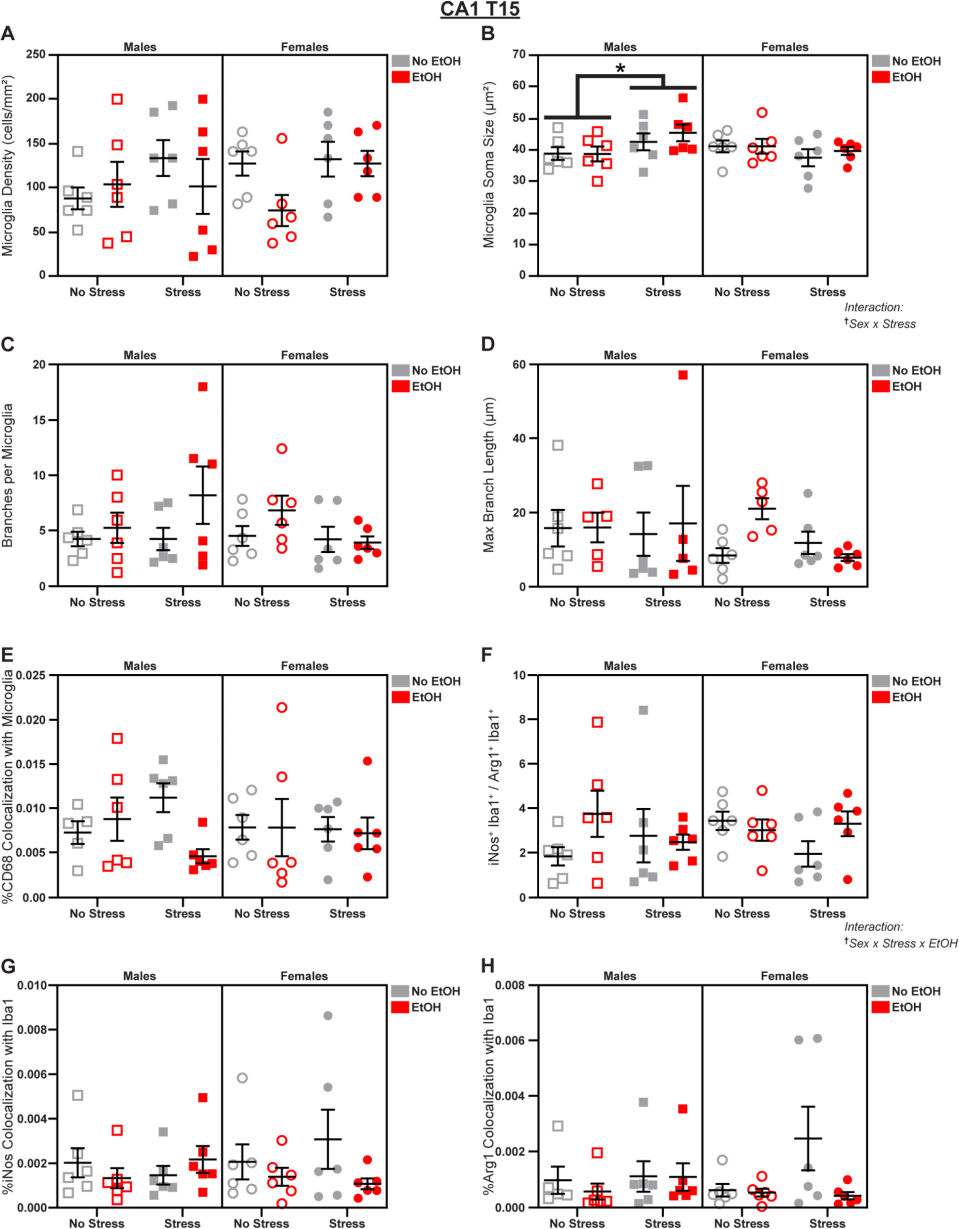
Microglial changes in CA1 at T15. Effects of sex, stress, and EtOH on **(A)** microglial density, **(B)** soma size, **(C)** branch number (1 outlier excluded), **(D)** branch length (4 outliers excluded), **(E)** CD68 expression (2 outliers excluded), **(F)** the iNos: Arg1 ratio, **(G)** iNos expression, and **(H)** Arg1 expression (1 outlier excluded). Text outside of graphs indicates two- or three-way interactions in 3-way ANOVAs, ^†^*p* < 0.05. Lines within graphs indicate main effects of stress in *post-hoc* sex-specific 2-way ANOVAs, *p < 0.05. *n* = 5–6 animals/group

We next examined microglial morphology in the HPC, which has been highlighted as another overlapping node in the circuits mediating stress and alcohol reactivity [54]. We characterized microglia separately in the three main subfields of the HPC: CA1, CA3, and the dentate gyrus (DG); plasticity in each of these areas is implicated in the development of substance use disorders [55]. In CA1, we observed interesting sex differences in the effects of stress and EtOH on microglial phenotypes at T15. There was a Sex x Stress interaction (3-way ANOVA: *F*_1,40_ = 5.739; *p* = 0.0214; *η*_*p*_^2^ = 0.1255; Fig. 6B) such that stress increased microglia soma size specifically in males (main effect of Stress in Male 2-way ANOVA: *F*_1,20_ = 4.543; *p* = 0.0456; *η*_*p*_^2^ = 0.1851; Fig. 6B; see Table S3 for further follow-ups). We also saw a Sex x Stress x EtOH interaction (3-way ANOVA: *F*_1,40_ = 4.120; *p* = 0.0491; *η*_*p*_^2^ = 0.0934; Fig. 6F; see Table S3 for further follow-ups) in the ratio of iNos: Arg1 levels, suggesting that stress decreased relative expression of iNos in females, and this was reversed by EtOH, but absolute levels of each protein’s colocalization with Iba1 were unaffected (Fig. 6G-H). There were no effects of sex, stress, or EtOH on microglial density (Fig. 6A), branch number (Fig. 6C), branch length (Fig. 6D), or CD68 expression (Fig. 6E); see Fig. S3 for representative micrographs and Table S3 for complete statistical analyses.

**Fig. 7:**
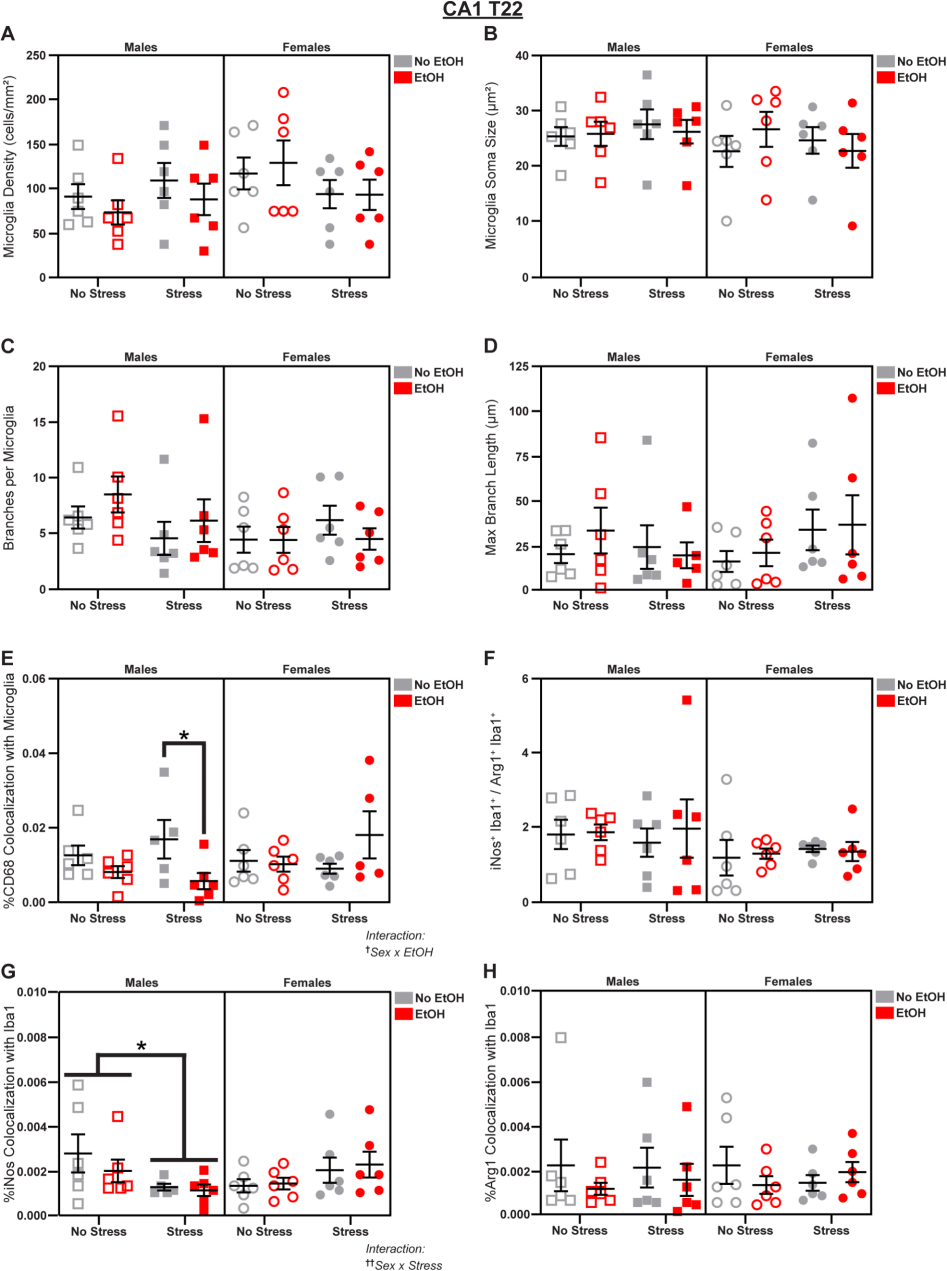
Microglial changes in CA1 at T22. Effects of sex, stress, and EtOH on **(A)** microglial density, **(B)** soma size, **(C)** branch number, **(D)** branch length (1 outlier excluded), **(E)** CD68 expression (2 outliers excluded), **(F)** the iNos: Arg1 ratio (1 outlier excluded), **(G)** iNos expression (1 outlier excluded), and **(H)** Arg1 expression. Text outside of graphs indicates two-way interactions in 3-way ANOVAs, ^†^*p* < 0.05, ^††^*p* < 0.01. Lines within graphs indicate main effects of stress in sex-specific 2-way ANOVAs or group differences in *post-hoc* Sidak’s multiple comparisons tests, *p < 0.05. *n* = 5–6 animals/group

At T22, we identified a Sex x EtOH interaction (3-way ANOVA: *F*_1,38_ = 7.100; *p* = 0.0113; *η*_*p*_^2^ = 0.1574; Fig. 7E) in microglial CD68 expression. *Post-hoc* analyses revealed a male-specific EtOH-induced decrease (main effect of EtOH in Male 2-way ANOVA: *F*_1,19_ = 6.971; *p* = 0.0161; *η*_*p*_^2^ = 0.2684; Fig. 7E) in this lysosomal marker, driven by the *Stress* group (Šídák’s t-test, Males Stress/No EtOH vs. Stress/EtOH: *t* = 2.604; *p* = 0.0345; *d* = 1.577; Fig. 7E). We also observed a Sex x Stress interaction (3-way ANOVA: *F*_1,39_ = 0.1673; *p* = 0.0079; *η*_*p*_^2^ = 0.1675; Fig. 7G) in microglial iNos expression in which stress decreased iNos in males (main effect of Stress in Male 2-way ANOVA: *F*_1,19_ = 4.803; *p* = 0.0411; *η*_*p*_^2^ = 0.2018; Fig. 7G; see Table S3 for further follow-ups), but Arg1 expression (Fig. 7H) and the iNos: Arg1 ratio were unaffected (Fig. 7F). There were no effects of sex, stress, or EtOH on microglial density (Fig. 7A), soma size (Fig. 7B), branch number (Fig. 7C), or branch length (Fig. 7D); see Fig. S3 for representative micrographs and Table S3 for complete statistical analyses.

These data reveal complex sex differences in CA1 microglia. In males, stress increased soma size, indicative of more reactive, ameboid morphology earlier in the paradigm; however, EtOH reduced CD68 expression, suggesting decreased lysosomal density at T22. We also saw sex differences in arginine metabolism: sex, stress, and EtOH altered the iNos: Arg1 ratio at T15 without affecting absolute levels of either enzyme, but stress decreased iNos expression at T22 in males without changing the ratio.

### Stress and EtOH have opposing effects in CA3

**Fig. 8:**
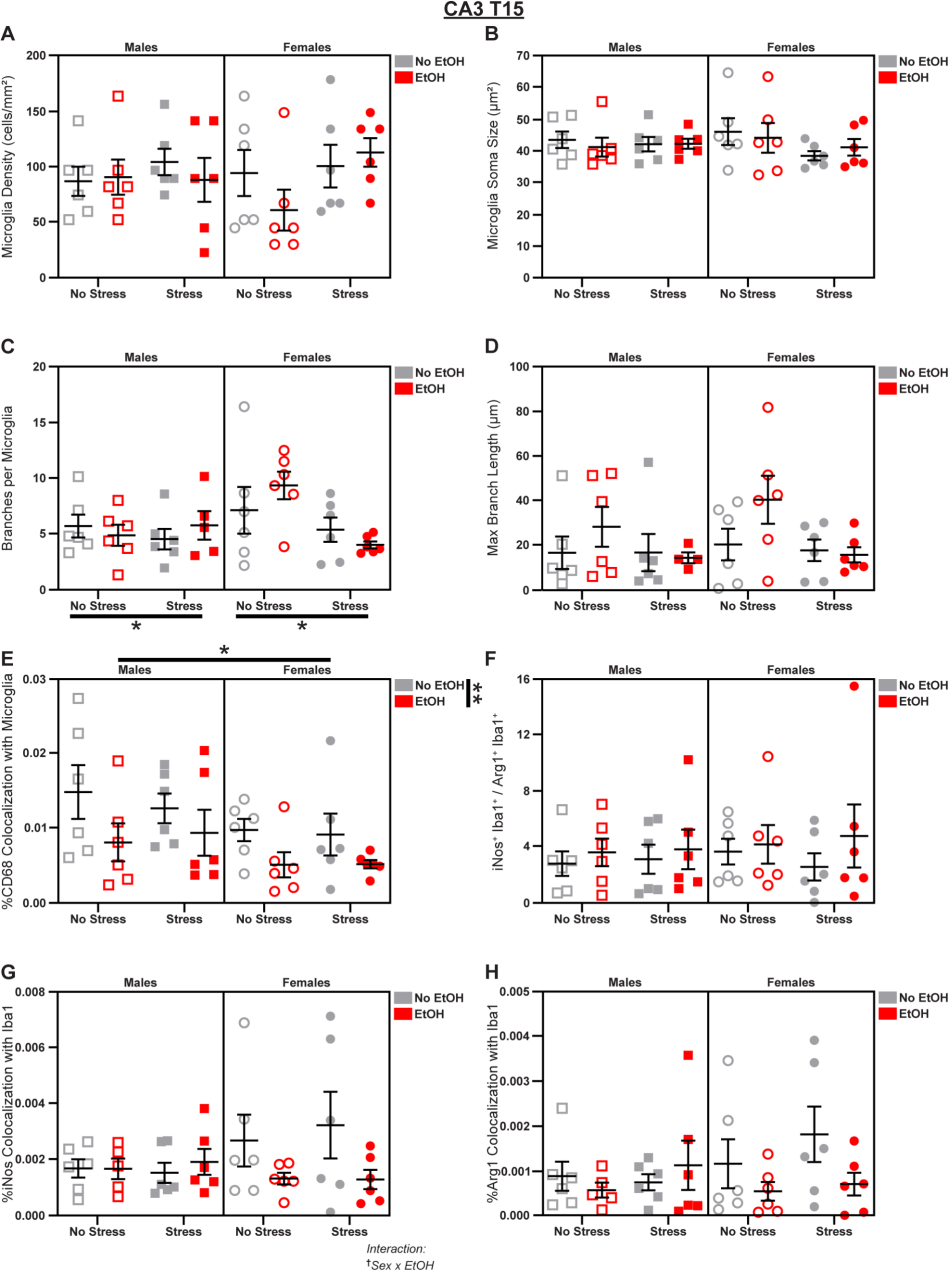
*Microglial changes in CA3 at T15.* Effects of sex, stress, and EtOH on **(A)** microglial density, **(B)** soma size, **(C)** branch number (1 outlier excluded). **(D)** branch length (2 outliers excluded), **(E)** CD68 expression, **(F)** the iNos: Arg1 ratio, **(G)** iNos expression (1 outlier excluded), and **(H)** Arg1 expression (1 outlier excluded). Text outside of graphs indicates two-way interactions in 3-way ANOVAs, ^†^*p* < 0.05. Lines outside of graphs indicate main effects of sex, stress, or EtOH in 3-way ANOVAs, *p < 0.05, **p < 0.01. *n* = 4–6 animals/group

Next, we assessed the CA3 subfield, which has a higher concentration of microglia compared to other hippocampal subregions [56]. On T15, we identified effects of sex, stress, and EtOH on microglial phenotypes. Stress reduced branch number (main effect of Stress in 3-way ANOVA: *F*_1,39_ = 4.647; *p* = 0.0373; *η*_*p*_^2^ = 0.1065; Fig. 8C). We also observed a sex effect in which males displayed increased colocalization of CD68 in microglia (main effect of Sex in 3-way ANOVA: *F*_1,40_ = 5.442; *p* = 0.0248; *η*_*p*_^2^ = 0.1197; Fig. 8E). There was a Sex x EtOH interaction (3-way ANOVA: *F*_1,39_ = 4.331; *p* = 0.0440; *η*_*p*_^2^ = 0.09995; Fig. 8G) in iNos expression, and *post-hoc* analyses revealed that EtOH decreased iNos colocalization specifically in females (main effect of EtOH in Female 2-way ANOVA: *F*_1,20_ = 4.482; *p* = 0.0470; *η*_*p*_^2^ = 0.1830; Fig. 8G; see Table S4 for further follow-ups). However, neither the iNos: Arg1 ratio (Fig. 8F) nor Arg1 expression (Fig. 8H) were altered. Additionally, EtOH decreased microglial CD68 expression (main effect of EtOH in 3-way ANOVA: *F*_1,40_ = 7.581; *p* = 0.0088; *η*_*p*_^2^ = 0.1593; Fig. 8E). There were no effects of sex, stress, or EtOH on microglial density (Fig. 8A), soma size (Fig. 8B), or branch length (Fig. 8D); see Fig. S4 for representative micrographs and Table S4 for complete statistical analyses.

**Fig. 9:**
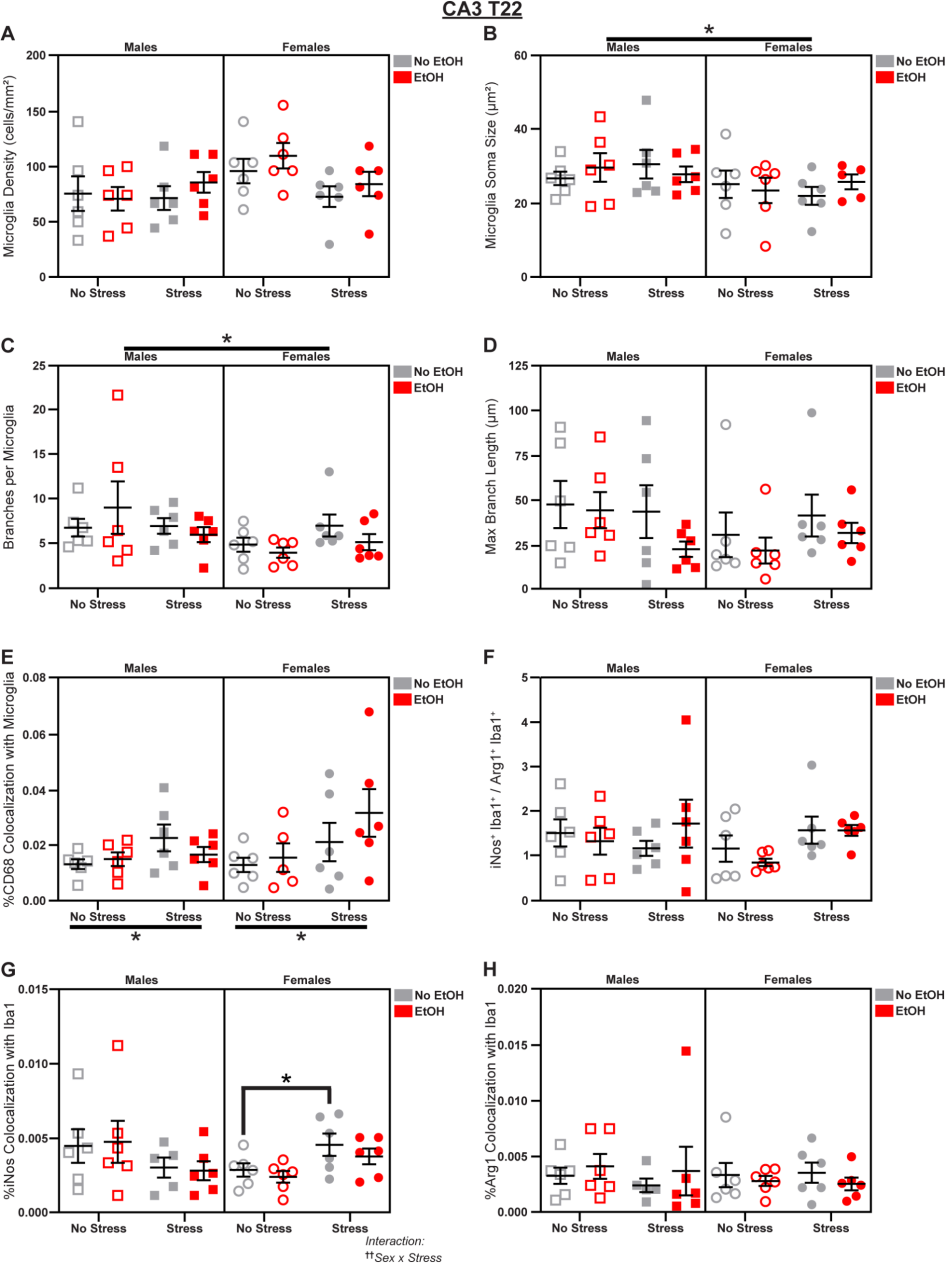
*Microglial changes in CA3 at T22.* Effects of sex, stress, and EtOH on **(A)** microglial density, **(B)** soma size (1 outlier excluded), **(C)** branch number, **(D)** branch length, **(E)** CD68 expression (1 outlier excluded), **(F)** the iNos: Arg1 ratio (1 outlier excluded), **(G)** iNos expression (1 outlier excluded), and **(H)** Arg1 expression (1 outlier excluded). Text outside of graphs indicates two-way interactions in 3-way ANOVAs, ^††^*p* < 0.01. Lines outside of graphs indicate main effects of sex or stress in 3-way ANOVAs, *p < 0.05. Lines within graphs indicate group differences in *post-hoc* Sidak’s multiple comparisons tests, *p < 0.05. *n* = 5–6 animals/group

Sex differences in soma size (main effect of Sex in 3-way ANOVA: *F*_1,39_ = 4.507; *p* = 0.0401; *η*_*p*_^2^ = 0.1036; Fig. 9B) and branch number (main effect of Sex in 3-way ANOVA: *F*_1,40_ = 4.178; *p* = 0.0476; *η*_*p*_^2^ = 0.0946; Fig. 9C) emerged by T22: both were increased in males compared to females. We also found that stress increased CD68 expression (main effect of Stress in 3-way ANOVA: *F*_1,39_ = 6.434; *p* = 0.0153; *η*_*p*_^2^ = 0.1416; Fig. 9E). We observed a Sex x Stress interaction (3-way ANOVA: *F*_1,39_ = 7.705; *p* = 0.0084; *η*_*p*_^2^ = 0.1650; Fig. 9G) in microglial iNos expression in which stress specifically increased iNos colocalization in females (main effect of Stress in Female 2-way ANOVA: *F*_1,20_ = 7.926; *p* = 0.0107; *η*_*p*_^2^ = 0.2838; Fig. 9G), and this was driven by the *No EtOH* group (Šídák’s t-test, Females No EtOH/No Stress vs. No EtOH/Stress: *t* = 2.195; *p* = 0.0401; *d* = 1.267; Fig. 9G). However, Arg1 expression (Fig. 9H) and the iNos: Arg1 ratio (Fig. 9F) were not changed. There were no effects of sex, stress, or EtOH on microglial density (Fig. 9A) or branch length (Fig. 9D); see Fig. S4 for representative micrographs and Table S4 for complete statistical analyses.

Microglia in CA3 were reactive to stress, exhibiting decreased ramification at T15 and increased lysosomal density at T22. In contrast, EtOH inhibited lysosomal density at the first timepoint. The sex differences in this subregion were complex, with males exhibiting increased lysosomal density, larger somas, and more ramification, and females showing enhanced susceptibility to oxidative stress induced by both EtOH and stress exposure.

### EtOH suppresses microglial reactivity in the DG

**Fig. 10:**
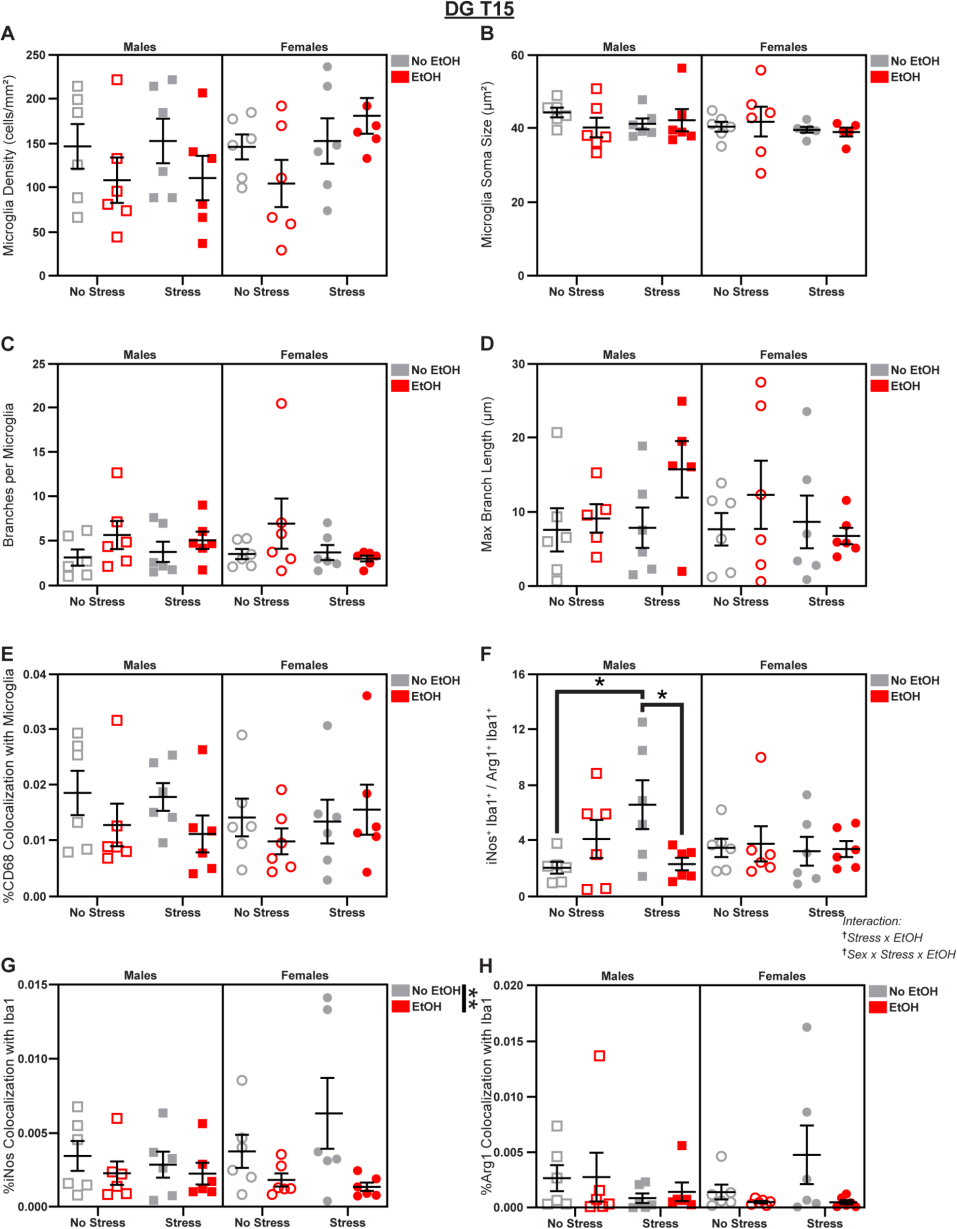
Microglial changes in the DG at T15. Effects of sex, stress, and EtOH on **(A)** microglial density (1 outlier excluded), **(B)** soma size (2 outliers excluded), **(C)** branch number, **(D)** branch length (2 outliers excluded), **(E)** CD68 expression, **(F)** the iNos: Arg1 ratio, **(G)** iNos expression, and **(H)** Arg1 expression. Text outside of graphs indicates two- or three-way interactions in 3-way ANOVAs, ^†^*p* < 0.05. Lines outside of graphs indicate main effects of EtOH in 3-way ANOVAs, **p < 0.01. Lines within graphs indicate group differences in *post-hoc* Sidak’s multiple comparisons tests, *p < 0.05. *n* = 5–6 animals/group

Finally, we measured microglial phenotypes in the DG, where stress-induced neuroinflammation contributes to depression symptomology [57]. We observed several interaction effects of sex, stress, and EtOH on microglia in the DG subregion. On T15, we saw a Stress x EtOH interaction (3-way ANOVA: *F*_1,40_ = 4.784; *p* = 0.0346; *η*_*p*_^2^ = 0.1068; Fig. 10F) and a Sex x Stress x EtOH interaction (3-way ANOVA: *F*_1,40_ = 4.422; *p* = 0.0418; *η*_*p*_^2^ = 0.0995; Fig. 10F) indicating male-specific changes to the iNos: Arg1 ratio (Stress x EtOH interaction in Male 2-way ANOVA: *F*_1,20_ = 7.579; *p* = 0.0123; *η*_*p*_^2^ = 0.2748; Fig. 10F). Stress increased relative iNos expression (Šídák’s t-test, Males No EtOH/No Stress vs. Males No EtOH/Stress: *t* = 2.787; *p* = 0.0226; *d* = 1.609; Fig. 10F), but this was reversed by EtOH exposure (Šídák’s t-test, Males Stress/No EtOH vs. Males Stress/EtOH: *t* = 2.620; *p* = 0.0325; *d* = 1.513; Fig. 10F). When measuring absolute differences in expression of these enzymes in microglia, we saw that EtOH decreased iNos colocalization (3-way ANOVA: *F*_1,40_ = 7.384; *p* = 0.0097; *η*_*p*_^2^ = 0.1559; Fig. 10G), but there were no changes to Arg1 (Fig. 10H). There were no effects of sex, stress, or EtOH on microglial density (Fig. 10A), soma size (Fig. 10B), branch number (Fig. 10C), branch length (Fig. 10D), or CD68 expression (Fig. 10E); see Fig. S5 for representative micrographs and Table S5 for complete statistical analyses.

**Fig. 11:**
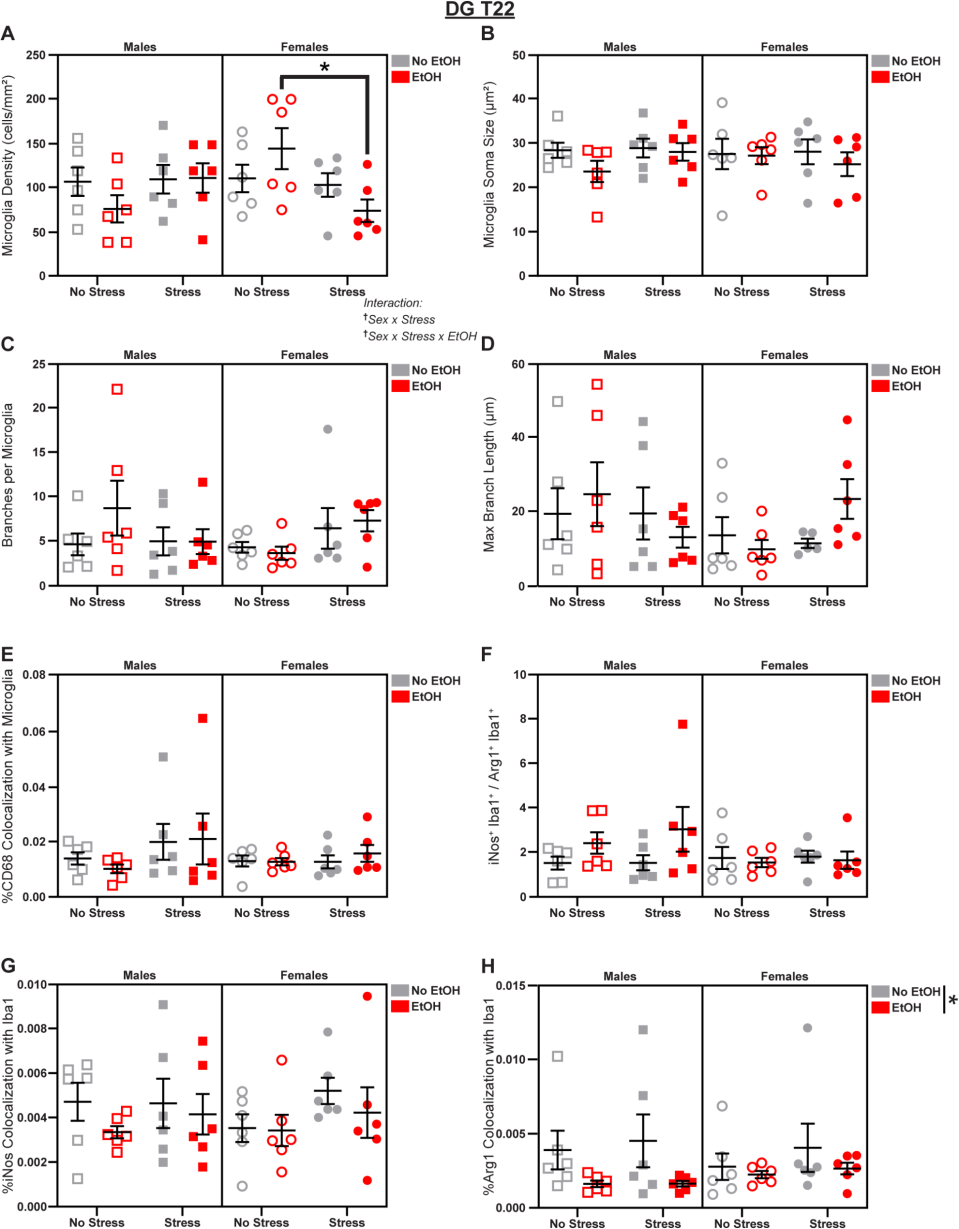
*Microglial changes in the DG at T22.* Effects of sex, stress, and EtOH on **(A)** microglial density, **(B)** soma size, **(C)** branch number, **(D)** branch length (1 outlier excluded), **(E)** CD68 expression, **(F)** the iNos: Arg1 ratio (1 outlier excluded), **(G)** iNos expression, and **(H)** Arg1 expression. Text outside of graphs indicates two- or three-way interactions in 3-way ANOVAs, ^†^*p* < 0.05. Lines outside of graphs indicate main effects of EtOH in 3-way ANOVAs, *p < 0.05. Lines within graphs indicate group differences in *post-hoc* Sidak’s multiple comparisons tests, *p < 0.05. *n* = 5–6 animals/group

On T22, we observed several interaction effects on microglial density. There was a Sex x Stress interaction (3-way ANOVA: *F*_1,40_ = 6.163; *p* = 0.0173; *η*_*p*_^2^ = 0.1335; Fig. 11A) and a Sex x Stress x EtOH interaction (3-way ANOVA: *F*_1,40_ = 4.187; *p* = 0.0473; *η*_*p*_^2^ = 0.0948; Fig. 11A). *Post-hoc* analyses revealed a female-specific stress-induced decrease in density (Stress effect in Female 2-way ANOVA: *F*_1,20_ = 5.369; *p* = 0.0312; *η*_*p*_^2^ = 0.2116; Fig. 11A) driven by the *EtOH* group (Šídák’s t-test, Females EtOH/No Stress vs. EtOH/Stress: *t* = 2.966; *p* = 0.0152; *d* = 1.712; Fig. 11A). We also found that EtOH reduced microglial Arg1 expression (main effect of EtOH in 3-way ANOVA: *F*_1,40_ = 5.828; *p* = 0.0204; *η*_*p*_^2^ = 0.1272; Fig. 11H), without altering expression of iNos (Fig. 11G) or the iNos: Arg1 ratio (Fig. 11F). There were no effects of sex, stress, or EtOH on soma size (Fig. 11B), branch number (Fig. 11C), branch length (Fig. 11D), or CD68 expression (Fig. 11E); see Fig. S5 for representative micrographs and Table S5 for complete statistical analyses.

These data reveal multi-faceted effects of EtOH. EtOH reversed the stress-induced increase in the iNos: Arg1 ratio in males and decreased absolute levels of iNos in all groups at T15, but then decreased Arg1 expression at T22. In stressed females at T22, EtOH decreased microglial density (Fig. 11G).

## Discussion

These studies show that brain microglial number and activation state are differentially altered by alcohol drinking and stress in male and female C57BL/6J mice. In addition, the intersecting effects of stress and EtOH on microglia are region-specific and time-dependent. In the amygdala, EtOH exerted a variety of suppressive effects on microglia at T15: cell density and CD68 expression were decreased in the BLA, and branch length was increased in the CeA. This may be surprising, as many studies have identified pro-inflammatory effects of EtOH [4], but others have observed downregulation of pro-inflammatory cytokines in the amygdala after repeated exposure to EtOH [58, 59].

Interestingly, these effects did not persist, and in fact, EtOH increased BLA cell density in males at T22, suggesting that early adaptive changes may be reversed following chronic alcohol exposure. This pattern has previously been observed in adolescent binge drinking models: shorter paradigms result in decreased microglial number, while longer paradigms have the opposite effect; unfortunately sex differences in these paradigms are not well-studied [30]. Clinical studies have reported increased expression of inflammatory markers such as GAS5, which regulates glucocorticoid signaling [60], and the chemokine MCP-1 [61], in the amygdala of alcohol-dependent subjects, but changes specifically to microglial markers were not seen [61], and these studies have not investigated sex differences.

We also observed sex differences in different brain regions and timepoints, independent of stress and EtOH exposure. In the amygdala, female microglia displayed morphology consistent with more reactive microglia, i.e. reduced branching and increased density. In CA3, female microglia exhibited decreased CD68 expression at T15 and decreased soma size and branch number at T22. These data reveal the highly dynamic nature of sex differences in microglia [62].

In the HPC, EtOH reduced microglial expression of the oxidative stress marker, iNos, in both CA3 and DG at T15. However, at T22, EtOH decreased DG levels of Arg1, the anti-inflammatory enzyme that competes with iNos for arginine metabolism [49]. This highlights how microglial reactivity to alcohol evolves with chronic use. Additionally, absolute levels of these enzymes were altered without changing the ratio of their expression relative to each other, further demonstrating that using one or both of these markers to dichotomize microglia into M1 vs. M2 categories may result in misrepresentation of their dynamics [23].

One consistent effect of EtOH was the downregulation of CD68 expression, which occurred in multiple regions of the HPC and amygdala and at multiple timepoints, suggesting a pattern of inhibited lysosomal activity which has been seen in other models of chronic EtOH exposure [63]. Although increased CD68 expression is typically associated with pro-inflammatory microglial activity and enhanced phagocytosis, and has been linked to engulfment of synapses following chronic stress [64], it remains debatable whether changes in CD68 represent protective destruction of damaged material or problematic engulfment of healthy structures without knowing the specific lysosomal contents involved [65–67].

Effects of stress on microglial phenotypes were restricted to the HPC and mainly skewed towards enhanced reactivity. In CA3, we observed stress-induced decreases in branch number at T15 and increases in CD68 expression at T22, regardless of sex. Some stress effects were specific to males, such as increased soma size in CA1 and relative iNos expression in DG at T15. In contrast, we observed a stress-induced decrease in absolute iNos expression in the male CA1 at T22. Additionally, some measures were downregulated by stress in females, including the iNos: Arg1 ratio in CA1 at T15 and density in DG at T22, whereas stress increased microglial iNos colocalization in females in CA3 at T22. This suggests more complex region specificity in females. Studies of neuronal plasticity have shown that CA3 neurons are highly sensitive to chronic stress, whereas CA1 neurons are less susceptible and may be further protected by estrogen release in females [68]; the estrogen receptors on microglia likely contribute to this neuroprotective effect [69, 70]. An alternative explanation is that this stress paradigm induces partial activation of microglia, an intermediate phenotype in which microglia exhibit some markers of classical activation but may in fact exert neuroprotective, rather than cytotoxic effects [71, 72]. The clinical literature has generally revealed pro-inflammatory effects of stress in the HPC, but has not investigated sex differences. Increased inflammation is associated with decreased HPC volume in people with post-traumatic stress disorder (PTSD) [73] and major depressive disorder (MDD) [74], and positron emission tomography (PET) has shown increased binding of TSPO, a marker of neuroimmune activation in the HPC of people with MDD [75].

The effects of EtOH are antagonistic to the effects of stress in some cases, and synergistic in others. EtOH reversed stress effects on the iNos: Arg1 ratio in the HPC of both sexes, but potentiated the female-specific reduction of microglia density. Repeated cycles of stress and alcohol exposure interact to drive chronic immune activation, as either can stimulate sensitization of microglia [14]; however, some research suggests that these patterns may be region-specific [76]. Clinical research on interactions between alcohol and stress in HPC immune function is scarce, and the data on alcohol alone is inconclusive and not differentiated by sex. PET data has shown increased TSPO binding in the HPC after acute alcohol consumption in social drinkers [77], but HPC TSPO is decreased in alcohol dependent subjects [78, 79]. Studies of postmortem brains have shown increased expression of inflammatory proteins [61, 80] and decreased glial cell density in the HPC of people with AUD [81].

Overall, effects of stress and EtOH were more prominent in the HPC than the amygdala, consistent with the observation that the HPC tends to display a unique vulnerability to stress and neuroinflammation, likely due to the high density of glucocorticoid receptors [82, 83], which are known to mediate inflammatory responses to stress and alcohol [83, 84]. Sex differences in the glucocorticoid-mediated vulnerability of the HPC may explain the pronounced cognitive and memory deficits seen in women and female rodents after binge drinking [85].

The current results reveal a complicated picture of microglial changes that are not consistently directional, i.e. diverging effects occur between different measures within the same brain region. Furthermore, these effects evolve over time in this paradigm, and it is important to note that the two timepoints chosen reflect differences in the type of acute stress exposure on the day of perfusion. Microglial changes observed on T22 represent a culmination of repeated stress and re-exposures to the stress context, with a full stress exposure on the day of perfusion, while T15 microglia experienced fewer stress exposures and acute re-exposure, which has been shown to elicit stress-conditioned responses, including escalated drinking [40, 86–88]. Therefore, these changes may represent neuroimmune adaptations to chronic stress or accumulating damage caused by persistent stress and EtOH exposure [30, 89].

By analyzing a variety of phenotypic markers, we demonstrate that the effects of stress and alcohol on microglia cannot be adequately captured by any single measure, and this is further complicated by sex differences. Thus, simply categorizing microglia into dichotomies such as M1 vs. M2 or active vs. inactive may obscure important distinctions in the ways microglia differ between sexes and how these differences lead to divergent reactions to stress and drug use. Furthermore, the changes we observed were nuanced, often with small effect sizes, complicated by interactions between variables and time- and subregion-specificity; thus, interpretations must be carefully constrained. Future research is needed to understand the functional and behavioral implications of these changes, particularly as they relate to stress-induced alcohol drinking. Additionally, further investigation into the molecular mechanisms driving the observed sex differences may provide targets for more effective AUD treatments in women.

### Perspectives and significance

This study established an analytical pipeline for comprehensive assessment of microglial phenotypes by combining quantification of cell density, morphology, and protein markers tied to inflammatory functions. While several studies have examined various aspects of microglial changes caused by alcohol consumption, few have incorporated stress exposure and very few have been designed to study sex differences. We have revealed nuanced effects of sex, stress, and alcohol exposure on microglia that cannot be captured by any single measure. Our results underscore the complexity of microglial contributions to the development of AUD, a disease which manifests differently in men and women and is highly comorbid with mood and stress disorders. Future research can apply this framework to characterize microglial changes in other brain regions, and investigate the potential of targeting microglia for the treatment of AUD.

## Conclusions

The present study has identified dynamic sex differences in microglia, both at baseline and in response to stress and alcohol, across several limbic brain regions. Our results demonstrate the heightened reactivity and sex-specificity of HPC microglia to stress and alcohol exposure. Chronic alcohol administration exerts several suppressive effects on microglia in the amygdala and on the lysosomal marker CD68, and can have both synergistic and antagonistic effects when combined with repeated stress. Our findings provide insight into the neuroimmune processes underlying the development of AUD in men and women, highlighting potential avenues for treatment.

## Electronic supplementary material

Below is the link to the electronic supplementary material.

Supplementary Material 1

## Data Availability

No datasets were generated or analysed during the current study.
