# Supplementary material for: Sex differences in the microglial response to stress and chronic alcohol exposure in mice
Source: Biol Sex Differ. 2025 Mar 4;16:19. doi: 10.1186/s13293-025-00701-y (PMC11881309; doi:10.1186/s13293-025-00701-y)
Supplement: Supplementary file 1 — Supplementary Material 1 [file 13293_2025_701_MOESM1_ESM.pdf]

Supplementary Materials

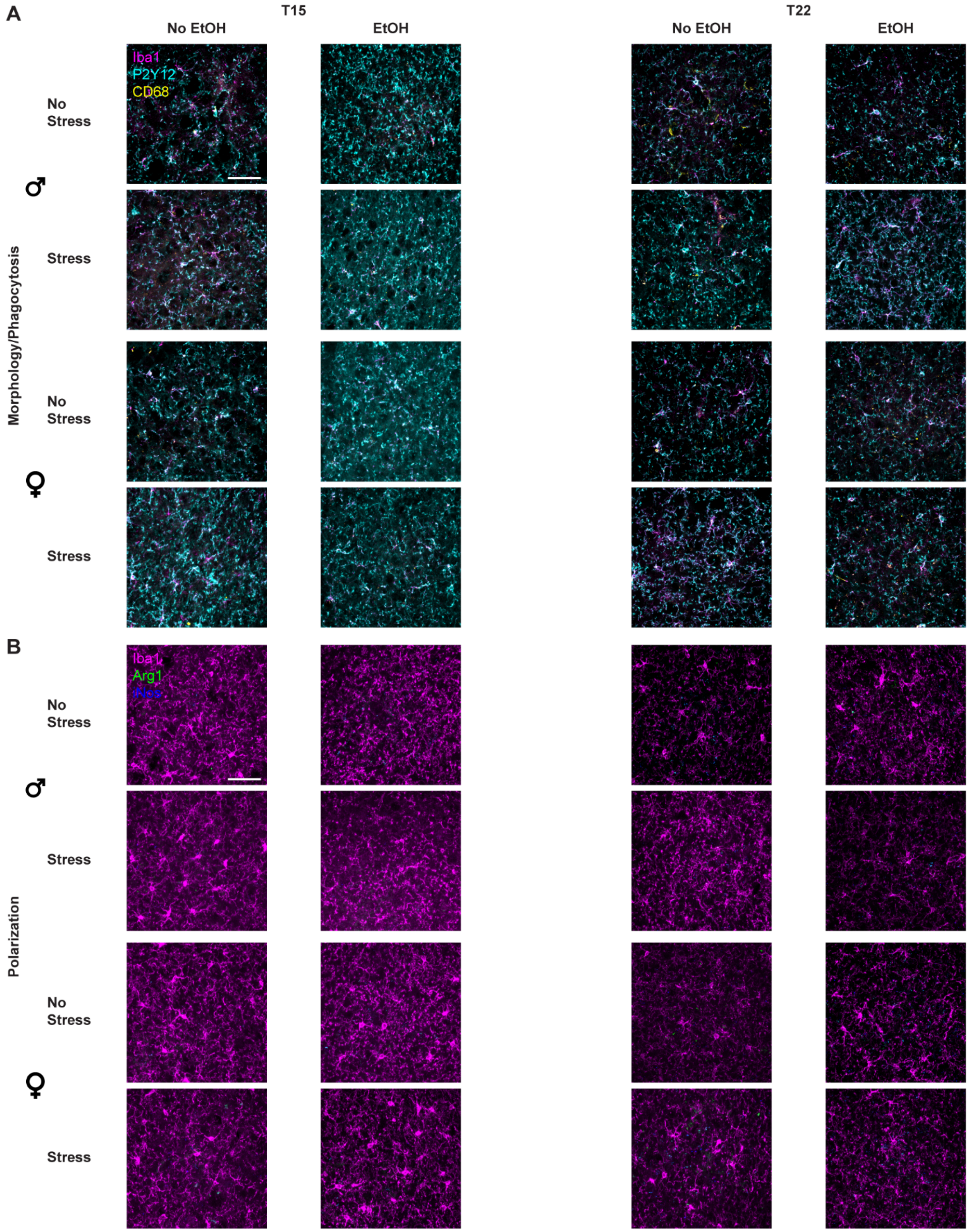

Fig. S1: Representative micrographs for BLA microglia.

Data shown in Figs. 2-3. All images are shown as Max Intensity Z-Projections. **A**. Representative images for the Morphology/Lysosome stain; Iba1 is magenta, P2Y12 is cyan, and CD68 is yellow. **B**. Representative images for the Polarization stain; Iba1 is magenta, Arg1 is green, and iNos is blue. Scale bar = 50 μm.

Table S1: Complete statistical analyses for characterization of BLA microglia.
Data shown in Figs. 2-3 and S1. *$p < 0.05$.

	**T15**		**T22**	
**Analysis**	***F / t***	***p***	***F / t***	***p***
3-way ANOVA: Microglia Density	Fig. 2A		Fig. 3A	
Effect of Sex	$F_{1,40}$ = 2.827	0.1005	$F_{1,40}$ = 0.1979	0.6588
Effect of Stress	$F_{1,40}$ = 2.246	0.1418	$F_{1,40}$ = 0.7380	0.3954
Effect of EtOH	$F_{1,40}$ = 5.776	0.0210*	$F_{1,40}$ = 0.3213	0.5740
Sex x Stress Interaction	$F_{1,40}$ = 1.448	0.2359	$F_{1,40}$ = 0.8070	0.3744
Sex x EtOH Interaction	$F_{1,40}$ = 0.09070	0.7648	$F_{1,40}$ = 5.425	0.0250*
Stress x EtOH Interaction	$F_{1,40}$ = 0.1668	0.6851	$F_{1,40}$ = 0.05048	0.8234
Sex x Stress x EtOH Interaction	$F_{1,40}$ = 6.278	0.0164*	$F_{1,40}$ = 2.990	0.0915
2-way ANOVA: Microglia Density, Males	Fig. 2A		Fig. 3A	
Effect of Stress	$F_{1,20}$ = 0.04283	0.8381	$F_{1,20}$ = 0.001002	0.2396
Effect of EtOH	$F_{1,20}$ = 3.598	0.0724	$F_{1,20}$ = 5.443	0.0302*
Stress x EtOH Interaction	$F_{1,20}$ = 4.178	0.0544	$F_{1,20}$ = 1.469	0.2396
Šídák's multiple comparisons test: Microglia Density, Males			Fig. 3A	
No Stress/No EtOH v. No Stress/EtOH			t = 0.7927	0.6833
Stress/No EtOH v. Stress/EtOH			t = 2.507	0.0414*
No EtOH/No Stress v. No EtOH/Stress			t = 0.8346	0.6563
EtOH/No Stress v. EtOH/Stress			t = 0.8794	0.6274
2-way ANOVA: Microglia Density, Females	Fig. 2A		Fig. 3A	
Effect of Stress	$F_{1,20}$ = 3.711	0.0684	$F_{1,20}$ = 1.256	0.2757
Effect of EtOH	$F_{1,20}$ = 2.246	0.1496	$F_{1,20}$ = 1.263	0.2744
Stress x EtOH Interaction	$F_{1,20}$ = 2.236	0.1505	$F_{1,20}$ = 1.552	0.2272

3-way ANOVA: Microglia Soma Size	Fig. 2B		Fig. 3B	
Effect of Sex	$F_{1,40}$ = 0.0228	0.8807	$F_{1,40}$ = 0.01323	0.9090
Effect of Stress	$F_{1,40}$ = 0.1240	0.7266	$F_{1,40}$ = 0.02411	0.8774
Effect of EtOH	$F_{1,40}$ = 0.4541	0.5043	$F_{1,40}$ = 0.01529	0.9022
Sex x Stress Interaction	$F_{1,40}$ = 0.5633	0.4573	$F_{1,40}$ = 1.308	0.2595
Sex x EtOH Interaction	$F_{1,40}$ = 0.3285	0.5697	$F_{1,40}$ = 0.01099	0.9170
Stress x EtOH Interaction	$F_{1,40}$ = 0.4078	0.5267	$F_{1,40}$ = 0.001190	0.9727
Sex x Stress x EtOH Interaction	$F_{1,40}$ = 0.09806	0.7558	$F_{1,40}$ = 0.8471	0.3629
3-way ANOVA: Branches per Microglia	Fig. 2C		Fig. 3C	
Effect of Sex	$F_{1,40}$ = 4.683	0.0365*	$F_{1,40}$ = 1.601	0.2131
Effect of Stress	$F_{1,40}$ = 0.4065	0.5274	$F_{1,40}$ = 0.9688	0.3309
Effect of EtOH	$F_{1,40}$ = 4.021	0.0517	$F_{1,40}$ = 0.2696	0.6065
Sex x Stress Interaction	$F_{1,40}$ = 1.079	0.3052	$F_{1,40}$ = 0.1434	0.7069
Sex x EtOH Interaction	$F_{1,40}$ = 1.117	0.2970	$F_{1,40}$ = 1.513	0.2259
Stress x EtOH Interaction	$F_{1,40}$ = 0.5978	0.440	$F_{1,40}$ = 0.06967	0.7932
Sex x Stress x EtOH Interaction	$F_{1,40}$ = 1.785	0.1891	$F_{1,40}$ = 2.521	0.1202
3-way ANOVA: Max Branch Length	Fig. 2D		Fig. 3D	
Effect of Sex	$F_{1,40}$ = 0.5019	0.4828	$F_{1,39}$ = 0.2489	0.6206
Effect of Stress	$F_{1,40}$ = 0.6967	0.4088	$F_{1,39}$ = 2.733	0.1063
Effect of EtOH	$F_{1,40}$ = 1.415	0.2413	$F_{1,39}$ = 3.269	0.0783
Sex x Stress Interaction	$F_{1,40}$ = 0.8789	0.3541	$F_{1,39}$ = 2.330	0.1350
Sex x EtOH Interaction	$F_{1,40}$ = 0.09262	0.7625	$F_{1,39}$ = 0.8869	0.3521
Stress x EtOH Interaction	$F_{1,40}$ = 0.05124	0.8221	$F_{1,39}$ = 0.0003573	0.9850
Sex x Stress x EtOH Interaction	$F_{1,40}$ = 2.526	0.1198	$F_{1,39}$ = 0.004653	0.9460
3-way ANOVA: %CD68 Colocalization with Microglia	Fig. 2E		Fig. 3E	

Effect of Sex	$F_{1,40}$ = 0.09250	0.7626	$F_{1,39}$ = 0.0008815	0.9765
Effect of Stress	$F_{1,40}$ = 0.03130	0.8605	$F_{1,39}$ = 0.7014	0.4074
Effect of EtOH	$F_{1,40}$ = 6.936	0.0120*	$F_{1,39}$ = 1.137	2.928
Sex x Stress Interaction	$F_{1,40}$ = 0.1541	0.6967	$F_{1,39}$ = 0.05490	0.8160
Sex x EtOH Interaction	$F_{1,40}$ = 0.1403	0.7099	$F_{1,39}$ = 0.08755	0.7689
Stress x EtOH Interaction	$F_{1,40}$ = 0.004071	0.9494	$F_{1,39}$ = 0.04733	0.8289
Sex x Stress x EtOH Interaction	$F_{1,40}$ = 0.02871	0.8663	$F_{1,39}$ = 0.002687	0.9589
3-way ANOVA: $iNos^{+}$ $Iba1^{+}$ / $Arg1^{+}$ $Iba1^{+}$	Fig. 2F		Fig. 3F	
Effect of Sex	$F_{1,40}$ = 0.06263	0.8037	$F_{1,39}$ = 0.6208	0.4355
Effect of Stress	$F_{1,40}$ = 0.9220	0.3427	$F_{1,39}$ = 2.710	0.1077
Effect of EtOH	$F_{1,40}$ = 3.656	0.0630	$F_{1,39}$ = 1.135	0.2933
Sex x Stress Interaction	$F_{1,40}$ = 0.02014	0.8879	$F_{1,39}$ = 1.146	0.2910
Sex x EtOH Interaction	$F_{1,40}$ = 0.2647	0.6097	$F_{1,39}$ = 0.04913	0.8257
Stress x EtOH Interaction	$F_{1,40}$ = 0.08034	0.7783	$F_{1,39}$ = 0.6369	0.4297
Sex x Stress x EtOH Interaction	$F_{1,40}$ = 2.309	0.1365	$F_{1,39}$ = 1.017	0.3194
3-way ANOVA: %iNos Colocalization with Iba1	Fig. 2G		Fig. 3G	
Effect of Sex	$F_{1,39}$ = 1.129	0.2945	$F_{1,40}$ = 1.728	0.1962
Effect of Stress	$F_{1,39}$ = 0.00004897	0.9945	$F_{1,40}$ = 2.778	0.1034
Effect of EtOH	$F_{1,39}$ = 0.3793	0.5416	$F_{1,40}$ = 0.07964	0.7792
Sex x Stress Interaction	$F_{1,39}$ = 4.455	0.0413*	$F_{1,40}$ = 0.4295	0.5160
Sex x EtOH Interaction	$F_{1,39}$ = 0.3086	0.5817	$F_{1,40}$ = 0.2438	0.6242
Stress x EtOH Interaction	$F_{1,39}$ = 0.2755	0.6026	$F_{1,40}$ = 0.7993	0.3767
Sex x Stress x EtOH Interaction	$F_{1,39}$ = 3.230	0.0800	$F_{1,40}$ = 0.2898	0.5934
2-way ANOVA: %iNos Colocalization with Iba1, Males	Fig. 2G			

Effect of Stress	$F_{1,20}$ = 1.623	0.2173		
Effect of EtOH	$F_{1,20}$ = 0.5032	0.4863		
Stress x EtOH Interaction	$F_{1,20}$ = 1.978	0.1750		
2-way ANOVA: %iNos Colocalization with Iba1, Females	Fig. 2G			
Effect of Stress	$F_{1,19}$ = 3.764	0.0674		
Effect of EtOH	$F_{1,19}$ = 0.003053	0.9565		
Stress x EtOH Interaction	$F_{1,19}$ = 1.359	0.2582		
3-way ANOVA: %Arg1 Colocalization with Iba1	Fig. 2H		Fig. 3H	
Effect of Sex	$F_{1,40}$ = 0.004179	0.9488	$F_{1,38}$ = 1.415	0.2416
Effect of Stress	$F_{1,40}$ = 0.9241	0.3422	$F_{1,38}$ = 3.862	0.0567
Effect of EtOH	$F_{1,40}$ = 1.302	0.2606	$F_{1,38}$ = 0.005287	0.9424
Sex x Stress Interaction	$F_{1,40}$ = 0.05860	0.8100	$F_{1,38}$ = 1.931	0.1728
Sex x EtOH Interaction	$F_{1,40}$ = 0.1276	0.7228	$F_{1,38}$ = 0.2322	0.6327
Stress x EtOH Interaction	$F_{1,40}$ = 0.4461	0.5080	$F_{1,38}$ = 0.7706	0.3856
Sex x Stress x EtOH Interaction	$F_{1,40}$ = 3.534	0.0674	$F_{1,38}$ = 1.466	0.2334

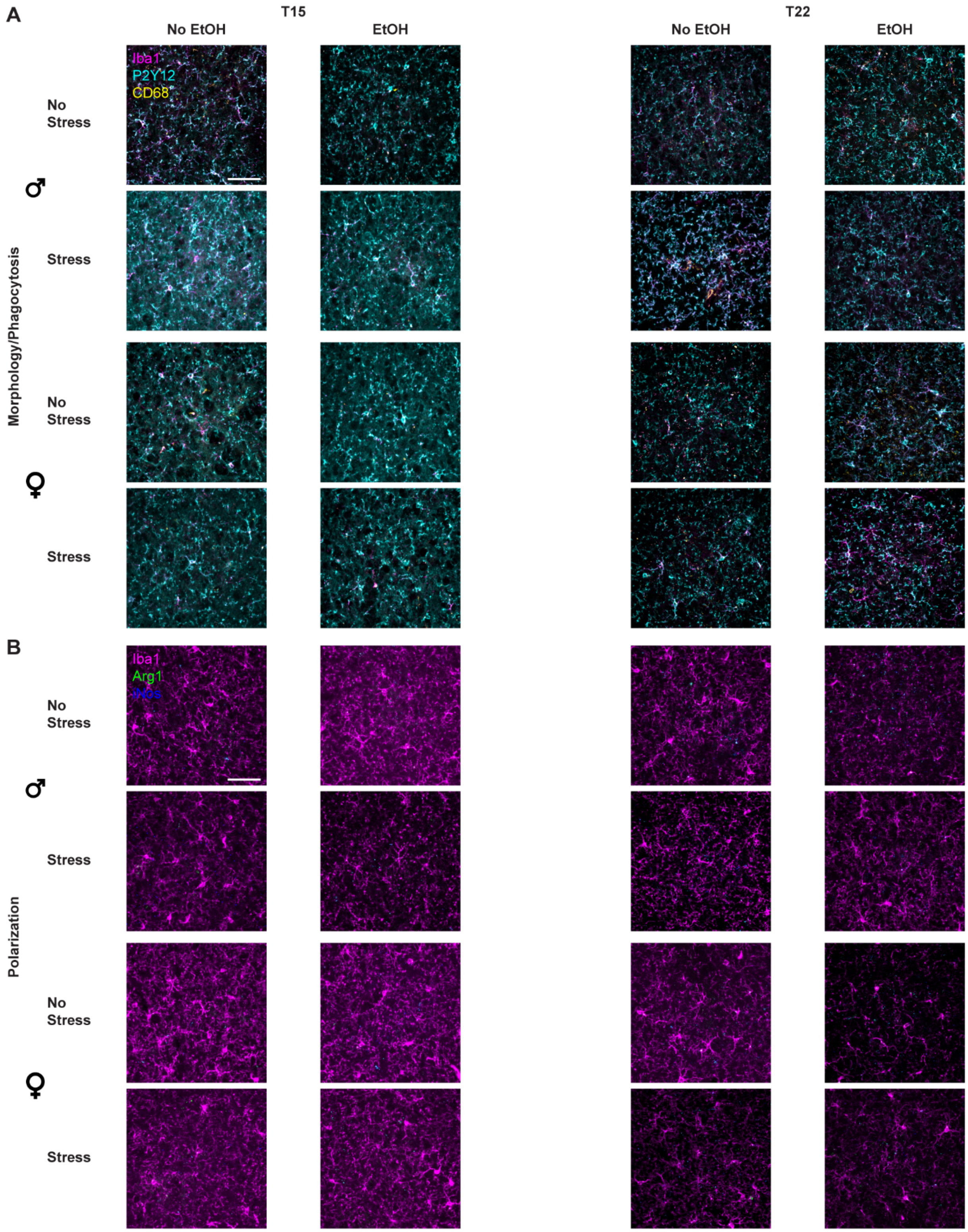

<u>Fig. S2: Representative micrographs for CeA microglia.</u>
Data shown in Figs. 4-5. All images are shown as Max Intensity Z-Projections. **A**. Representative images for the Morphology/Lysosome stain; Iba1 is magenta, P2Y12 is

cyan, and CD68 is yellow. **B**. Representative images for the Polarization stain; Iba1 is magenta, Arg1 is green, and iNos is blue. Scale bar = 50 µm.

Table S2: Complete statistical analyses for characterization of CeA microglia. Data shown in Figs. 4-5 and S2. *$p < 0.05$; **$p < 0.01$.

	**T15**		**T22**	
**Analysis**	***F / t***	***p***	***F / t***	***p***
3-way ANOVA: Microglia Density	Fig. 4A		Fig. 5A	
Effect of Sex	$F_{1,40}$ = 0.2003	0.6569	$F_{1,40}$ = 8.986	0.0047**
Effect of Stress	$F_{1,40}$ = 0.2487	0.6208	$F_{1,40}$ = 0.01877	0.8917
Effect of EtOH	$F_{1,40}$ = 2.315	0.1360	$F_{1,40}$ = 0.2865	0.5955
Sex x Stress Interaction	$F_{1,40}$ = 0.01746	0.8955	$F_{1,40}$ = 0.1664	0.6855
Sex x EtOH Interaction	$F_{1,40}$ = 1.734	0.1954	$F_{1,40}$ = 0.01974	0.8890
Stress x EtOH Interaction	$F_{1,40}$ = 1.919	0.1737	$F_{1,40}$ = 0.6193	0.4359
Sex x Stress x EtOH Interaction	$F_{1,40}$ = 2.420	0.1277	$F_{1,40}$ = 0.06705	0.7970
3-way ANOVA: Microglia Soma Size	Fig. 4B		Fig. 5B	
Effect of Sex	$F_{1,40}$ = 0.007658	0.9307	$F_{1,40}$ = 1.818	0.1852
Effect of Stress	$F_{1,40}$ = 0.02466	0.8760	$F_{1,40}$ = 2.250	0.1415
Effect of EtOH	$F_{1,40}$ = 0.1873	0.6675	$F_{1,40}$ = 0.002722	0.9587
Sex x Stress Interaction	$F_{1,40}$ = 0.08601	0.7708	$F_{1,40}$ = 0.06918	0.7939
Sex x EtOH Interaction	$F_{1,40}$ = 0.3238	0.5725	$F_{1,40}$ = 0.09593	0.7584
Stress x EtOH Interaction	$F_{1,40}$ = 1.063	0.3087	$F_{1,40}$ = 1.435	0.2380
Sex x Stress x EtOH Interaction	$F_{1,40}$ = 0.7935	0.3784	$F_{1,40}$ = 0.9271	0.3414
3-way ANOVA: Branches per Microglia	Fig. 4C		Fig. 5C	
Effect of Sex	$F_{1,40}$ = 0.2268	0.6365	$F_{1,40}$ = 7.910	0.0076**
Effect of Stress	$F_{1,40}$ = 0.01084	0.9176	$F_{1,40}$ = 0.05415	0.8172

Effect of EtOH	$F_{1,40}$ = 2.481	0.1231	$F_{1,40}$ = 0.001023	0.9746
Sex x Stress Interaction	$F_{1,40}$ = 0.7438	0.3936	$F_{1,40}$ = 0.0002639	0.9871
Sex x EtOH Interaction	$F_{1,40}$ = 1.885	0.1774	$F_{1,40}$ = 0.2593	0.6134
Stress x EtOH Interaction	$F_{1,40}$ = 0.09391	0.7608	$F_{1,40}$ = 0.3519	0.5564
Sex x Stress x EtOH Interaction	$F_{1,40}$ = 1.713	0.1981	$F_{1,40}$ = 0.00417	0.9473
3-way ANOVA: Max Branch Length	Fig. 4D		Fig. 5D	
Effect of Sex	$F_{1,40}$ = 0.004799	0.9451	$F_{1,39}$ = 0.5371	0.0258*
Effect of Stress	$F_{1,40}$ = 0.001569	0.9686	$F_{1,39}$ = 0.02632	0.8720
Effect of EtOH	$F_{1,40}$ = 4.727	0.0357*	$F_{1,39}$ = 0.2620	0.6117
Sex x Stress Interaction	$F_{1,40}$ = 2.479	0.1232	$F_{1,39}$ = 0.002680	0.9590
Sex x EtOH Interaction	$F_{1,40}$ = 1.351	0.2520	$F_{1,39}$ = 0.4144	0.5235
Stress x EtOH Interaction	$F_{1,40}$ = 1.231	0.2738	$F_{1,39}$ = 0.1276	0.7229
Sex x Stress x EtOH Interaction	$F_{1,40}$ = 0.5198	0.4751	$F_{1,39}$ = 0.2882	0.5944
3-way ANOVA: %CD68 Colocalization with Microglia	Fig. 4E		Fig. 5E	
Effect of Sex	$F_{1,38}$ = 0.001451	0.9698	$F_{1,40}$ = 0.1337	0.7166
Effect of Stress	$F_{1,38}$ = 2.592	0.1157	$F_{1,40}$ = 0.007200	0.9328
Effect of EtOH	$F_{1,38}$ = 3.463	0.0705	$F_{1,40}$ = 0.2066	0.6519
Sex x Stress Interaction	$F_{1,38}$ = 0.8684	0.3573	$F_{1,40}$ = 0.5560	0.4602
Sex x EtOH Interaction	$F_{1,38}$ = 1.673	0.2036	$F_{1,40}$ = 0.004394	0.9475
Stress x EtOH Interaction	$F_{1,38}$ = 2.935	0.0948	$F_{1,40}$ = 0.9009	0.3482
Sex x Stress x EtOH Interaction	$F_{1,38}$ = 0.8739	0.3558	$F_{1,40}$ = 2.936	0.0944
3-way ANOVA: iNos$^+$ Iba1$^+$ / Arg1$^+$ Iba1$^+$	Fig. 4F		Fig. 5F	
Effect of Sex	$F_{1,40}$ = 0.5891	0.4473	$F_{1,38}$ = 0.02071	0.8863

Effect of Stress	$F_{1,40}$ = 2.518	0.1204	$F_{1,38}$ = 0.2375	0.6288
Effect of EtOH	$F_{1,40}$ = 0.004818	0.9450	$F_{1,38}$ = 0.7223	0.4007
Sex x Stress Interaction	$F_{1,40}$ = 0.02018	0.8878	$F_{1,38}$ = 2.187	0.1474
Sex x EtOH Interaction	$F_{1,40}$ = 0.4629	0.5002	$F_{1,38}$ = 3.405	0.0728
Stress x EtOH Interaction	$F_{1,40}$ = 0.001456	0.9698	$F_{1,38}$ = 0.013818	0.9092
Sex x Stress x EtOH Interaction	$F_{1,40}$ = 0.04654	0.8303	$F_{1,38}$ = 0.1227	0.7281
3-way ANOVA: %iNos Colocalization with Iba1	Fig. 4G		Fig. 5G	
Effect of Sex	$F_{1,39}$ = 0.1259	0.7247	$F_{1,40}$ = 0.2053	0.6529
Effect of Stress	$F_{1,39}$ = 0.5862	0.4485	$F_{1,40}$ = 4.047	0.0510
Effect of EtOH	$F_{1,39}$ = 0.2128	0.6471	$F_{1,40}$ = 0.9758	0.3292
Sex x Stress Interaction	$F_{1,39}$ = 1.036	0.3149	$F_{1,40}$ = 1.665	0.2043
Sex x EtOH Interaction	$F_{1,39}$ = 0.2209	0.6410	$F_{1,40}$ = 1.427	0.2393
Stress x EtOH Interaction	$F_{1,39}$ = 0.4402	0.5109	$F_{1,40}$ = 0.2253	0.6376
Sex x Stress x EtOH Interaction	$F_{1,39}$ = 3.089	0.0867	$F_{1,40}$ = 1.322	0.2570
3-way ANOVA: %Arg1 Colocalization with Iba1	Fig. 4H		Fig. 5H	
Effect of Sex	$F_{1,39}$ = 0.002577	0.9598	$F_{1,40}$ = 0.2842	0.5969
Effect of Stress	$F_{1,39}$ = 0.1119	0.7398	$F_{1,40}$ = 4.063	0.0506
Effect of EtOH	$F_{1,39}$ = 0.1295	0.7209	$F_{1,40}$ = 0.4185	0.5214
Sex x Stress Interaction	$F_{1,39}$ = 2.529	0.1199	$F_{1,40}$ = 0.08430	0.7731
Sex x EtOH Interaction	$F_{1,39}$ = 0.3733	0.5448	$F_{1,40}$ = 0.06673	0.7975
Stress x EtOH Interaction	$F_{1,39}$ = 0.001331	0.9711	$F_{1,40}$ = 1.115	0.2974
Sex x Stress x EtOH Interaction	$F_{1,39}$ = 1.040	0.3141	$F_{1,40}$ = 0.2617	0.6118

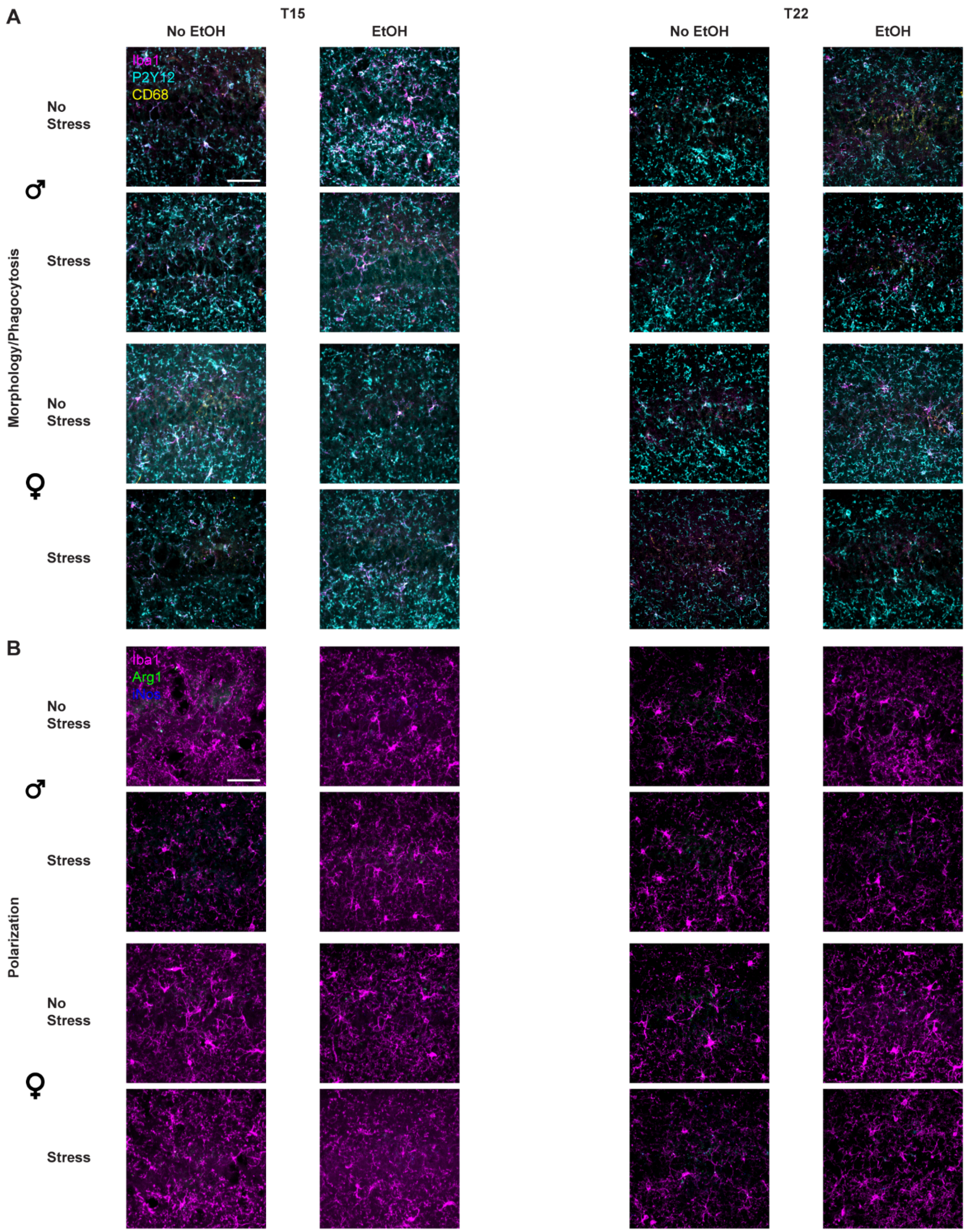

<u>Fig. S3: Representative micrographs for CA1 microglia.</u>

Data shown in Figs. 6-7. All images are shown as Max Intensity Z-Projections. **A.** Representative images for the Morphology/Lysosome stain; Iba1 is magenta, P2Y12 is

cyan, and CD68 is yellow. **B**. Representative images for the Polarization stain; Iba1 is magenta, Arg1 is green, and iNos is blue. Scale bar = 50 μm.

Table S3: Complete statistical analyses for characterization of CA1 microglia.
Data shown in Figs. 6-7 and S3. *p < 0.05.

	**T15**		**T22**	
**Analysis**	***F / t***	***p***	***F / t***	***p***
3-way ANOVA: Microglia Density	Fig. 6A		Fig. 7A	
Effect of Sex	$F_{1,40}$ = 0.3647	0.5488	$F_{1,40}$ = 1.977	0.1674
Effect of Stress	$F_{1,40}$ = 3.136	0.0842	$F_{1,40}$ = 0.2721	0.6048
Effect of EtOH	$F_{1,40}$ = 1.679	0.2025	$F_{1,40}$ = 0.2982	0.5881
Sex x Stress Interaction	$F_{1,40}$ = 0.06717	0.7968	$F_{1,40}$ = 3.282	0.0776
Sex x EtOH Interaction	$F_{1,40}$ = 0.5392	0.4671	$F_{1,40}$ = 0.9961	0.3243
Stress x EtOH Interaction	$F_{1,40}$ = 1.846e-014	>0.9999	$F_{1,40}$ = 0.1017	0.7515
Sex x Stress x EtOH Interaction	$F_{1,40}$ = 2.838	0.0999	$F_{1,40}$ = 0.03313	0.8565
3-way ANOVA: Microglia Soma Size	Fig. 6B		Fig. 7B	
Effect of Sex	$F_{1,40}$ = 0.8704	0.3564	$F_{1,40}$ = 1.291	0.2625
Effect of Stress	$F_{1,40}$ = 0.6902	0.4110	$F_{1,40}$ = 0.008168	0.9284
Effect of EtOH	$F_{1,40}$ = 0.5732	0.4534	$F_{1,40}$ = 0.02822	0.8674
Sex x Stress Interaction	$F_{1,40}$ = 5.739	0.0214*	$F_{1,40}$ = 0.3882	0.5368
Sex x EtOH Interaction	$F_{1,40}$ = 0.007586	0.9310	$F_{1,40}$ = 0.1634	0.6882
Stress x EtOH Interaction	$F_{1,40}$ = 0.6302	0.4320	$F_{1,40}$ = 1.135	0.2931
Sex x Stress x EtOH Interaction	$F_{1,40}$ = 0.01932	0.8901	$F_{1,40}$ = 0.3199	0.5748
2-way ANOVA: Microglia Soma Size, Males	Fig. 6B			
Effect of Stress	$F_{1,20}$ = 4.543	0.0456*		
Effect of EtOH	$F_{1,20}$ = 0.3110	0.5832		
Stress x EtOH Interaction	$F_{1,20}$ = 0.3797	0.5447		
Šídák's multiple comparisons test: Microglia Soma Size, Males	Fig. 6B			

No Stress/No EtOH v. No Stress/EtOH	t = 0.04139	0.9989		
Stress/No EtOH v. Stress/EtOH	t = 0.8301	0.6593		
No EtOH/No Stress v. No EtOH/Stress	t = 1.071	0.5054		
EtOH/No Stress v. EtOH/Stress	t = 1.943	0.1281		
2-way ANOVA: Microglia Soma Size, Females	Fig. 6B			
Effect of Stress	$F_{1,20}$ = 1.433	0.2452		
Effect of EtOH	$F_{1,20}$ = 0.2628	0.6138		
Stress x EtOH Interaction	$F_{1,20}$ = 0.2510	0.6219		
3-way ANOVA: Branches per Microglia	Fig. 6C		Fig. 7C	
Effect of Sex	$F_{1,40}$ = 0.4230	0.5192	$F_{1,40}$ = 2.512	0.1209
Effect of Stress	$F_{1,40}$ = 0.005900	0.9392	$F_{1,40}$ = 0.3860	0.5379
Effect of EtOH	$F_{1,40}$ = 3.431	0.0714	$F_{1,40}$ = 0.2549	0.6164
Sex x Stress Interaction	$F_{1,40}$ = 2.682	0.1093	$F_{1,40}$ = 2.460	0.1247
Sex x EtOH Interaction	$F_{1,40}$ = 0.6160	0.4372	$F_{1,40}$ = 1.959	0.1693
Stress x EtOH Interaction	$F_{1,40}$ = 0.007748	0.9303	$F_{1,40}$ = 0.3119	0.5796
Sex x Stress x EtOH Interaction	$F_{1,40}$ = 2.154	0.1500	$F_{1,40}$ = 0.09718	0.7569
3-way ANOVA: Max Branch Length	Fig. 6D		Fig. 7D	
Effect of Sex	$F_{1,37}$ = 1.051	0.3119	$F_{1,39}$ = 0.1128	0.7388
Effect of Stress	$F_{1,37}$ = 0.5785	0.4517	$F_{1,39}$ = 0.6162	0.4372
Effect of EtOH	$F_{1,37}$ = 0.7525	0.3913	$F_{1,39}$ = 0.2892	0.5938
Sex x Stress Interaction	$F_{1,37}$ = 0.4718	0.4964	$F_{1,39}$ = 2.057	0.1595
Sex x EtOH Interaction	$F_{1,37}$ = 0.1723	0.6805	$F_{1,39}$ = 0.0007983	0.9776
Stress x EtOH Interaction	$F_{1,37}$ = 1.048	0.3126	$F_{1,39}$ = 0.4326	0.5146
Sex x Stress x EtOH Interaction	$F_{1,37}$ = 2.055	0.1601	$F_{1,39}$ = 0.2749	0.6030
3-way ANOVA: %CD68 Colocalization with Microglia	Fig. 6E		Fig. 7E	

Effect of Sex	$F_{1,39}$ = 0.06064	0.8068	$F_{1,38}$ = 0.3313	0.5683
Effect of Stress	$F_{1,39}$ = 0.04270	0.8374	$F_{1,38}$ = 0.7376	0.3958
Effect of EtOH	$F_{1,39}$ = 1.056	0.3104	$F_{1,38}$ = 0.7009	0.4077
Sex x Stress Interaction	$F_{1,39}$ = 0.01309	0.9095	$F_{1,38}$ = 0.1877	0.6673
Sex x EtOH Interaction	$F_{1,39}$ = 0.7188	0.4017	$F_{1,38}$ = 7.100	0.0113*
Stress x EtOH Interaction	$F_{1,39}$ = 2.509	0.1213	$F_{1,38}$ = 0.1301	0.7203
Sex x Stress x EtOH Interaction	$F_{1,39}$ = 2.023	0.1629	$F_{1,38}$ = 3.455	0.0708
2-way ANOVA: %CD68 Colocalization with Microglia, Males			Fig. 7E	
Effect of Stress			$F_{1,19}$ = 0.1030	0.7518
Effect of EtOH			$F_{1,19}$ = 6.971	0.0161*
Stress x EtOH Interaction			$F_{1,19}$ = 1.276	0.2728
Šídák's multiple comparisons test: %CD68 Colocalization with Microglia, Males			Fig. 7E	
No Stress/No EtOH v. No Stress/EtOH			t = 1.095	0.4921
Stress/No EtOH v. Stress/EtOH			t = 2.604	0.0345*
No EtOH/No Stress v. No EtOH/Stress			t = 1.002	0.5497
EtOH/No Stress v. EtOH/Stress			t = 0.5858	0.8107
2-way ANOVA: %CD68 Colocalization with Microglia, Females			Fig. 7E	
Effect of Stress			$F_{1,19}$ = 0.7449	0.3989
Effect of EtOH			$F_{1,19}$ = 1.490	0.2371
Stress x EtOH Interaction			$F_{1,19}$ = 2.198	0.1546
3-way ANOVA: $iNos^+ Iba1^+$ / $Arg1^+ Iba1^+$	Fig. 6F		Fig. 7F	
Effect of Sex	$F_{1,40}$ = 0.1981	0.6586	$F_{1,40}$ = 3.073	0.0873
Effect of Stress	$F_{1,40}$ = 0.6327	0.4311	$F_{1,40}$ = 0.02317	0.8798

Effect of EtOH	$F_{1,40}$ = 1.697	0.2002	$F_{1,40}$ = 0.1769	0.6763
Sex x Stress Interaction	$F_{1,40}$ = 0.1915	0.6640	$F_{1,40}$ = 0.1379	0.7123
Sex x EtOH Interaction	$F_{1,40}$ = 0.1235	0.7272	$F_{1,40}$ = 0.1220	0.7288
Stress x EtOH Interaction	$F_{1,40}$ = 0.04656	0.8303	$F_{1,40}$ = 0.01493	0.9033
Sex x Stress x EtOH Interaction	$F_{1,40}$ = 4.120	0.0491*	$F_{1,40}$ = 0.1993	0.6577
2-way ANOVA: $iNos^+$ $Iba1^+$ / $Arg1^+$ $Iba1^+$, Males	Fig. 6F			
Effect of Stress	$F_{1,20}$ = 0.04373	0.8365		
Effect of EtOH	$F_{1,20}$ = 0.9346	0.3452		
Stress x EtOH Interaction	$F_{1,20}$ = 1.723	0.2042		
2-way ANOVA: $iNos^+$ $Iba1^+$ / $Arg1^+$ $Iba1^+$, Females	Fig. 6F			
Effect of Stress	$F_{1,20}$ = 1.417	0.2479		
Effect of EtOH	$F_{1,20}$ = 0.8431	0.3695		
Stress x EtOH Interaction	$F_{1,20}$ = 3.066	0.0953		
3-way ANOVA: %iNos Colocalization with Iba1	Fig. 6G		Fig. 7G	
Effect of Sex	$F_{1,40}$ = 0.1017	0.7514	$F_{1,39}$ = 0.005792	0.9397
Effect of Stress	$F_{1,40}$ = 0.2571	0.6149	$F_{1,39}$ = 0.3479	0.5587
Effect of EtOH	$F_{1,40}$ = 1.855	0.1808	$F_{1,39}$ = 0.1673	0.6847
Sex x Stress Interaction	$F_{1,40}$ = 0.04503	0.8330	$F_{1,39}$ = 7.849	0.0079**
Sex x EtOH Interaction	$F_{1,40}$ = 1.918	0.1738	$F_{1,39}$ = 0.8254	0.3692
Stress x EtOH Interaction	$F_{1,40}$ = 0.001248	0.9720	$F_{1,39}$ = 0.3204	0.5746
Sex x Stress x EtOH Interaction	$F_{1,40}$ = 1.977	0.1675	$F_{1,39}$ = 0.1239	0.7267
2-way ANOVA: %iNos Colocalization with Iba1, Males			Fig. 7G	
Effect of Stress			$F_{1,19}$ = 4.803	0.0411*
Effect of EtOH			$F_{1,19}$ = 0.7249	0.4051
Stress x EtOH Interaction			$F_{1,19}$ = 0.3519	0.5600

Šídák's multiple comparisons test: %iNos Colocalization with Iba1, Males			Fig. 7G	
No Stress/No EtOH v. No Stress/EtOH			t = 1.047	0.3083
Stress/No EtOH v. Stress/EtOH			t = 0.1784	0.8603
No EtOH/No Stress v. No EtOH/Stress			t = 1.924	0.0695
EtOH/No Stress v. EtOH/Stress			t = 1.158	0.2612
2-way ANOVA: %iNos Colocalization with Iba1, Females			Fig. 7G	
Effect of Stress			$F_{1,20}$ = 2.986	0.0994
Effect of EtOH			$F_{1,20}$ = 0.1523	0.7005
Stress x EtOH Interaction			$F_{1,20}$ = 0.02797	0.8689
3-way ANOVA: %Arg1 Colocalization with Iba1	Fig. 6H		Fig. 7H	
Effect of Sex	$F_{1,39}$ = 0.03618	0.8501	$F_{1,40}$ = 0.007092	0.9333
Effect of Stress	$F_{1,39}$ = 2.523	0.1203	$F_{1,40}$ = 0.002553	0.9600
Effect of EtOH	$F_{1,39}$ = 2.882	0.0975	$F_{1,40}$ = 1.021	0.3183
Sex x Stress Interaction	$F_{1,39}$ = 0.5125	0.4783	$F_{1,40}$ = 0.06383	0.8018
Sex x EtOH Interaction	$F_{1,39}$ = 1.280	0.2648	$F_{1,40}$ = 0.3676	0.5477
Stress x EtOH Interaction	$F_{1,39}$ = 1.090	0.3029	$F_{1,40}$ = 0.8896	0.3512
Sex x Stress x EtOH Interaction	$F_{1,39}$ = 2.415	0.1282	$F_{1,40}$ = 0.1918	0.6638

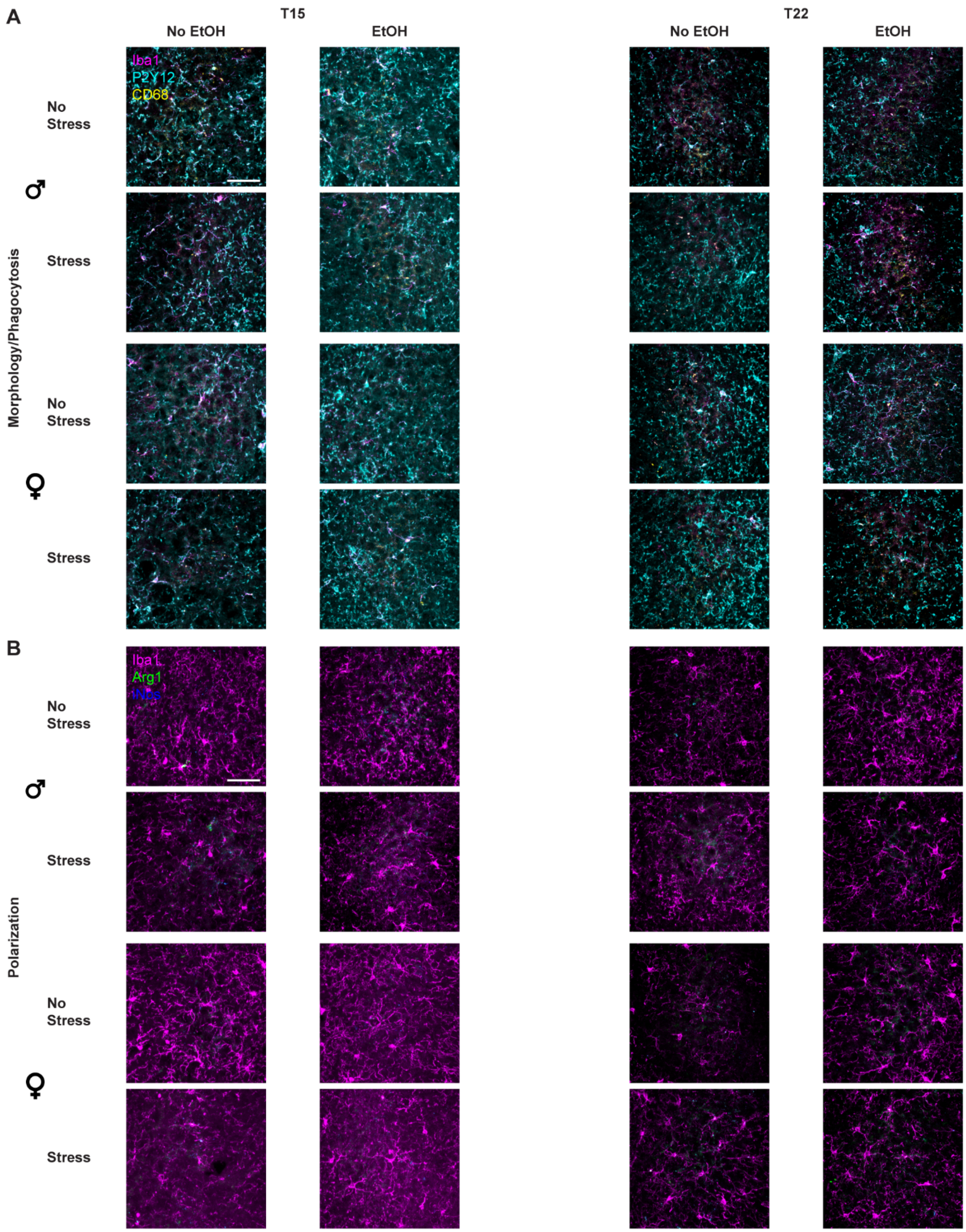

Fig. S4: Representative micrographs for CA3 microglia.

Data shown in Figs. 8-9. All images are shown as Max Intensity Z-Projections. **A**. Representative images for the Morphology/Lysosome stain; Iba1 is magenta, P2Y12 is

cyan, and CD68 is yellow. **B**. Representative images for the Polarization stain; Iba1 is magenta, Arg1 is green, and iNos is blue. Scale bar = 50 µm.

Table S4: Complete statistical analyses for characterization of CA3 microglia.
Data shown in Figs. 8-9 and S4. *$p < 0.05$; **$p < 0.01$.

	**T15**		**T22**	
**Analysis**	***F / t***	***p***	***F / t***	***p***
3-way ANOVA: Microglia Density	Fig. 8A		Fig. 9A	
Effect of Sex	$F_{1,40}$ = 0.0006745	0.9794	$F_{1,40}$ = 3.389	0.0730
Effect of Stress	$F_{1,40}$ = 2.348	0.1333	$F_{1,40}$ = 1.406	0.2428
Effect of EtOH	$F_{1,40}$ = 0.4917	0.4872	$F_{1,40}$ = 1.186	0.2826
Sex x Stress Interaction	$F_{1,40}$ = 0.8262	0.3688	$F_{1,40}$ = 3.461	0.0702
Sex x EtOH Interaction	$F_{1,40}$ = 0.03305	0.8567	$F_{1,40}$ = 0.2416	0.6257
Stress x EtOH Interaction	$F_{1,40}$ = 0.2975	0.5885	$F_{1,40}$ = 0.2610	0.6123
Sex x Stress x EtOH Interaction	$F_{1,40}$ = 1.895	0.1763	$F_{1,40}$ = 0.4423	0.5098
3-way ANOVA: Microglia Soma Size	Fig. 8B		Fig. 9B	
Effect of Sex	$F_{1,40}$ = 0.006672	0.9353	$F_{1,39}$ = 4.507	0.0401*
Effect of Stress	$F_{1,40}$ = 1.661	0.2049	$F_{1,39}$ = 0.01782	0.8945
Effect of EtOH	$F_{1,40}$ = 0.02800	0.8680	$F_{1,39}$ = 0.06973	0.7931
Sex x Stress Interaction	$F_{1,40}$ = 1.482	0.2306	$F_{1,39}$ = 0.1079	0.7443
Sex x EtOH Interaction	$F_{1,40}$ = 0.1182	0.7328	$F_{1,39}$ = 0.04457	0.8339
Stress x EtOH Interaction	$F_{1,40}$ = 0.6810	0.4141	$F_{1,39}$ = 0.0004148	0.9839
Sex x Stress x EtOH Interaction	$F_{1,40}$ = 0.06622	0.7982	$F_{1,39}$ = 1.647	0.2069
3-way ANOVA: Branches per Microglia	Fig. 8C		Fig. 9C	
Effect of Sex	$F_{1,39}$ = 2.134	0.1521	$F_{1,40}$ = 4.178	0.0476*
Effect of Stress	$F_{1,39}$ = 4.647	0.0373*	$F_{1,40}$ = 0.01562	0.9012
Effect of EtOH	$F_{1,39}$ = 0.1377	0.7126	$F_{1,40}$ = 0.1537	0.6971
Sex x Stress Interaction	$F_{1,39}$ = 3.972	0.0533	$F_{1,40}$ = 2.637	0.1122

Sex x EtOH Interaction	$F_{1,39}$ = 0.01717	0.8964	$F_{1,40}$ = 1.127	0.2949
Stress x EtOH Interaction	$F_{1,39}$ = 0.2004	0.6569	$F_{1,40}$ = 1.219	0.2761
Sex x Stress x EtOH Interaction	$F_{1,39}$ = 2.720	0.1071	$F_{1,40}$ = 0.3580	0.5530
3-way ANOVA: Max Branch Length	Fig. 8D		Fig. 9D	
Effect of Sex	$F_{1,40}$ = 0.7343	0.3969	$F_{1,40}$ = 1.192	0.2814
Effect of Stress	$F_{1,40}$ = 3.731	0.0609	$F_{1,40}$ = 0.02974	0.8639
Effect of EtOH	$F_{1,40}$ = 1.655	0.2061	$F_{1,40}$ = 2.067	0.1583
Sex x Stress Interaction	$F_{1,40}$ = 0.4035	0.5291	$F_{1,40}$ = 2.425	0.1273
Sex x EtOH Interaction	$F_{1,40}$ = 0.1710	0.6815	$F_{1,40}$ = 0.03629	0.8499
Stress x EtOH Interaction	$F_{1,40}$ = 2.869	0.0985	$F_{1,40}$ = 0.4011	0.5301
Sex x Stress x EtOH Interaction	$F_{1,40}$ = 0.1448	0.7056	$F_{1,40}$ = 0.3214	0.5739
3-way ANOVA: %CD68 Colocalization with Microglia	Fig. 8E		Fig. 9E	
Effect of Sex	$F_{1,40}$ = 5.442	0.0248*	$F_{1,39}$ = 9.9685	0.3311
Effect of Stress	$F_{1,40}$ = 0.04512	0.8329	$F_{1,39}$ = 6.434	0.0153*
Effect of EtOH	$F_{1,40}$ = 7.581	0.0088**	$F_{1,39}$ = 0.4048	0.5283
Sex x Stress Interaction	$F_{1,40}$ = 0.003077	0.9560	$F_{1,39}$ = 0.8974	0.3493
Sex x EtOH Interaction	$F_{1,40}$ = 0.04127	0.8400	$F_{1,39}$ = 1.529	0.2237
Stress x EtOH Interaction	$F_{1,40}$ = 0.3798	0.5412	$F_{1,39}$ = 1.824e-005	0.9966
Sex x Stress x EtOH Interaction	$F_{1,40}$ = 0.1705	0.6818	$F_{1,39}$ = 1.257	0.2691
3-way ANOVA: $iNos^+$ $Iba1^+$ / $Arg1^+$ $Iba1^+$	Fig. 8F		Fig. 9F	
Effect of Sex	$F_{1,40}$ = 0.2493	0.6203	$F_{1,40}$ = 0.4670	0.4983
Effect of Stress	$F_{1,40}$ = 0.0001753	0.9895	$F_{1,40}$ = 2.003	0.1648
Effect of EtOH	$F_{1,40}$ = 1.319	0.2576	$F_{1,40}$ = 0.004019	0.9498
Sex x Stress Interaction	$F_{1,40}$ = 0.07739	0.7823	$F_{1,40}$ = 1.665	0.2043

Sex x EtOH Interaction	$F_{1,40}$ = 0.1066	0.7457	$F_{1,40}$ = 0.6681	0.4185
Stress x EtOH Interaction	$F_{1,40}$ = 0.1849	0.6695	$F_{1,40}$ = 1.564	0.2183
Sex x Stress x EtOH Interaction	$F_{1,40}$ = 0.2382	0.6282	$F_{1,40}$ = 0.2689	0.6069
3-way ANOVA: %iNos Colocalization with Iba1	Fig. 8G		Fig. 9G	
Effect of Sex	$F_{1,39}$ = 0.9525	0.3351	$F_{1,39}$ = 0.4011	0.5302
Effect of Stress	$F_{1,39}$ = 0.1216	0.7292	$F_{1,39}$ = 0.01875	0.8918
Effect of EtOH	$F_{1,39}$ = 2.744	0.1057	$F_{1,39}$ = 0.2550	0.6164
Sex x Stress Interaction	$F_{1,39}$ = 0.05892	0.8095	$F_{1,39}$ = 7.705	0.0084**
Sex x EtOH Interaction	$F_{1,39}$ = 4.331	0.0440*	$F_{1,39}$ = 0.3191	0.5754
Stress x EtOH Interaction	$F_{1,39}$ = 0.01026	0.9198	$F_{1,39}$ = 0.1211	0.7297
Sex x Stress x EtOH Interaction	$F_{1,39}$ = 0.3110	0.5803	$F_{1,39}$ = 0.005933	0.9390
2-way ANOVA: %iNos Colocalization with Iba1, Males	Fig. 8G		Fig. 9G	
Effect of Stress	$F_{1,19}$ = 0.01476	0.9046	$F_{1,19}$ = 2.623	0.1218
Effect of EtOH	$F_{1,19}$ = 0.2370	0.6319	$F_{1,19}$ = 0.001110	0.9738
Stress x EtOH Interaction	$F_{1,19}$ = 0.2737	0.6069	$F_{1,19}$ = 0.05587	0.8157
2-way ANOVA: %iNos Colocalization with Iba1, Females	Fig. 8G		Fig. 9G	
Effect of Stress	$F_{1,20}$ = 0.1122	0.7411	$F_{1,20}$ = 7.926	0.0107*
Effect of EtOH	$F_{1,20}$ = 4.482	0.0470*	$F_{1,20}$ = 1.303	0.2672
Stress x EtOH Interaction	$F_{1,20}$ = 0.1393	0.7129	$F_{1,20}$ = 0.08360	0.7755
Šídák's multiple comparisons test: %iNos Colocalization with Iba1, Females	Fig. 8G		Fig. 9G	
No Stress/No EtOH v. No Stress/EtOH	*t* = 1.233	0.4099	*t* = 0.6026	0.5536
Stress/No EtOH v. Stress/EtOH	*t* = 1.761	0.1783	*t* = 1.011	0.3239
No EtOH/No Stress v. No EtOH/Stress	*t* =0.5008	0.8571	*t* = 2.195	0.0401*

EtOH/No Stress v. EtOH/Stress	*t* = 0.02703	0.9995	*t* = 1.786	0.0892
3-way ANOVA: %Arg1 Colocalization with Iba1	Fig. 8H		Fig. 9H	
Effect of Sex	$F_{1,39}$ = 0.6256	0.4338	$F_{1,39}$ = 0.1610	0.6904
Effect of Stress	$F_{1,39}$ = 1.159	0.2883	$F_{1,39}$ = 0.1806	0.6732
Effect of EtOH	$F_{1,39}$ = 2.095	0.1558	$F_{1,39}$ = 0.03851	0.8454
Sex x Stress Interaction	$F_{1,39}$ = 0.1217	0.7290	$F_{1,39}$ = 0.1552	0.6957
Sex x EtOH Interaction	$F_{1,39}$ = 2.455	0.1252	$F_{1,39}$ = 1.379	0.2474
Stress x EtOH Interaction	$F_{1,39}$ = 0.02931	0.8649	$F_{1,39}$ = 0.0002369	0.9878
Sex x Stress x EtOH Interaction	$F_{1,39}$ = 1.080	0.3051	$F_{1,39}$ = 0.08366	0.7739

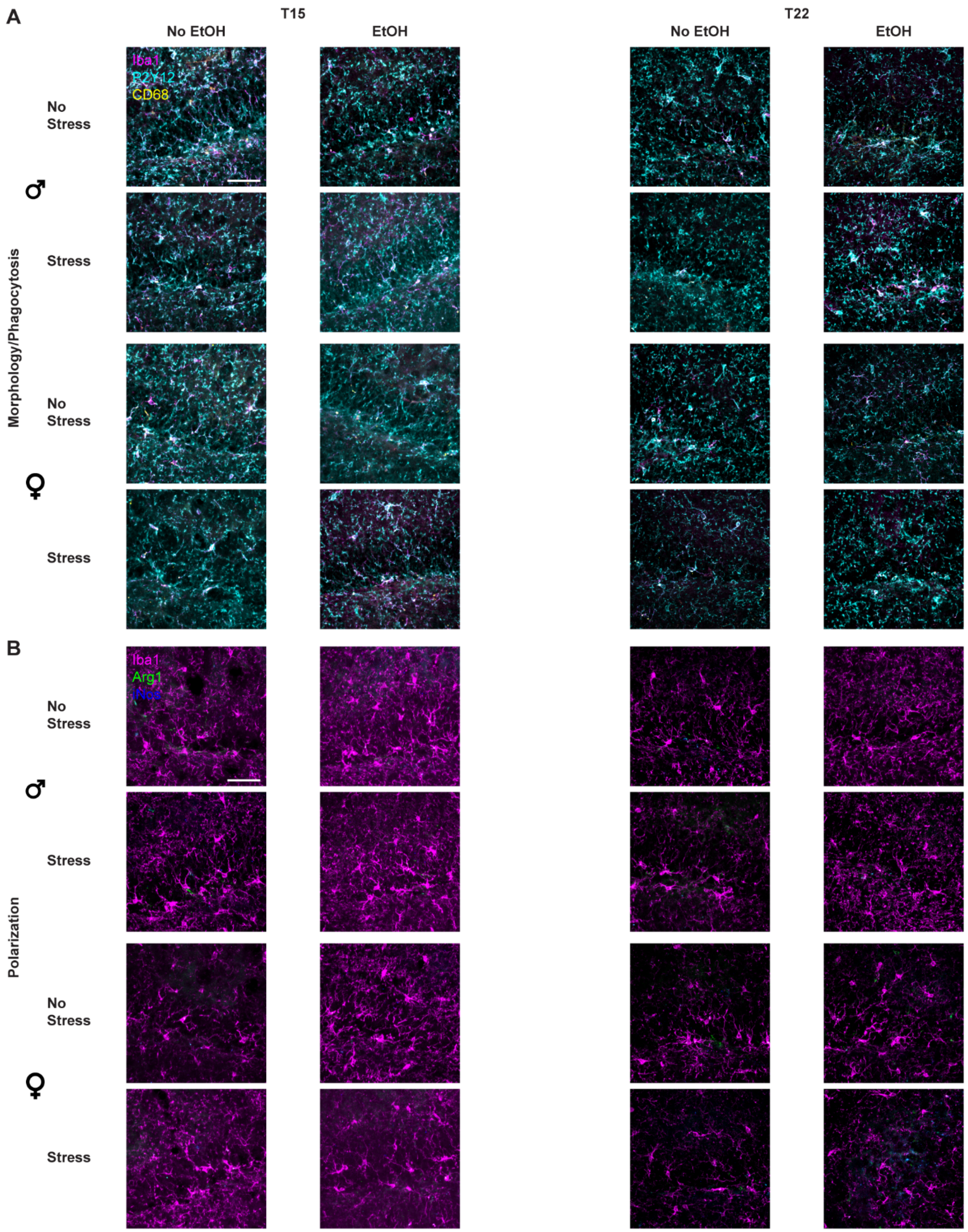

<u>Fig. S5: Representative micrographs for DG microglia.</u>

Data shown in Figs. 10-11. All images are shown as Max Intensity Z-Projections. **A**. Representative images for the Morphology/Lysosome stain; Iba1 is magenta, P2Y12 is

cyan, and CD68 is yellow. **B**. Representative images for the Polarization stain; Iba1 is magenta, Arg1 is green, and iNos is blue. Scale bar = 50 µm.

Table S5: Complete statistical analyses for characterization of DG microglia. Data shown in Figs. 10-11 and S5. **p* < 0.05; ***p* < 0.01.

	**T15**		**T22**	
**Analysis**	***F / t***	***p***	***F / t***	***p***
3-way ANOVA: Microglia Density	Fig. 10A		Fig. 11A	
Effect of Sex	$F_{1,40}$ = 0.9625	0.3324	$F_{1,40}$ = 0.3730	0.5449
Effect of Stress	$F_{1,40}$ = 1.866	0.1795	$F_{1,40}$ = 0.7428	0.3939
Effect of EtOH	$F_{1,40}$ = 1.917	0.1739	$F_{1,40}$ = 0.2824	0.5981
Sex x Stress Interaction	$F_{1,40}$ = 1.231	0.2739	$F_{1,40}$ = 6.163	0.0173*
Sex x EtOH Interaction	$F_{1,40}$ = 0.9989	0.3236	$F_{1,40}$ = 0.5324	0.4698
Stress x EtOH Interaction	$F_{1,40}$ = 0.9625	0.3324	$F_{1,40}$ = 0.4403	0.5108
Sex x Stress x EtOH Interaction	$F_{1,40}$ = 1.191	0.2817	$F_{1,40}$ = 4.187	0.0473*
2-way ANOVA: Microglia Density, Males			Fig. 11A	
Effect of Stress			$F_{1,20}$ = 1.370	0.2555
Effect of EtOH			$F_{1,20}$ = 0.8297	0.3732
Stress x EtOH Interaction			$F_{1,20}$ = 0.9974	0.3299
2-way ANOVA: Microglia Density, Females			Fig. 11A	
Effect of Stress			$F_{1,20}$ = 5.369	0.0312*
Effect of EtOH			$F_{1,20}$ = 0.01887	0.8921
Stress x EtOH Interaction			$F_{1,20}$ = 3.525	0.0751
Šídák's multiple comparisons test: Microglia Density, Females			Fig. 11A	
No Stress/No EtOH v. No Stress/EtOH			*t* = 1.425	0.3105
Stress/No EtOH v. Stress/EtOH			*t* = 1.230	0.4114
No EtOH/No Stress v. No EtOH/Stress			*t* = 0.3108	0.9420
EtOH/No Stress v. EtOH/Stress			*t* = 2.966	0.0152*

3-way ANOVA: Microglia Soma Size	Fig. 10B		Fig. 11B	
Effect of Sex	$F_{1,39}$ = 1.227	0.2748	$F_{1,40}$ = 0.01689	0.8972
Effect of Stress	$F_{1,39}$ = 0.5510	0.4623	$F_{1,40}$ = 0.2511	0.6191
Effect of EtOH	$F_{1,39}$ = 0.1288	0.7216	$F_{1,40}$ = 1.634	0.2085
Sex x Stress Interaction	$F_{1,39}$ = 0.1554	0.6956	$F_{1,40}$ = 0.8585	0.3597
Sex x EtOH Interaction	$F_{1,39}$ = 0.3472	0.5591	$F_{1,40}$ = 0.1253	0.7252
Stress x EtOH Interaction	$F_{1,39}$ = 0.2268	0.6365	$F_{1,40}$ = 0.04536	0.8324
Sex x Stress x EtOH Interaction	$F_{1,39}$ = 1.188	0.2825	$F_{1,40}$ = 0.8513	0.3617
3-way ANOVA: Branches per Microglia	Fig. 10C		Fig. 11C	
Effect of Sex	$F_{1,40}$ = 0.01359	0.9078	$F_{1,40}$ = 0.1096	0.7423
Effect of Stress	$F_{1,40}$ = 0.9415	0.3377	$F_{1,40}$ = 0.2419	0.6255
Effect of EtOH	$F_{1,40}$ = 2.944	0.0939	$F_{1,40}$ = 0.7710	0.3851
Sex x Stress Interaction	$F_{1,40}$ = 0.9994	0.3235	$F_{1,40}$ = 3.683	0.0621
Sex x EtOH Interaction	$F_{1,40}$ = 0.08172	0.7765	$F_{1,40}$ = 0.6376	0.4293
Stress x EtOH Interaction	$F_{1,40}$ = 1.936	0.1718	$F_{1,40}$ = 0.2888	0.5940
Sex x Stress x EtOH Interaction	$F_{1,40}$ = 0.5562	0.4601	$F_{1,40}$ = 1.366	0.2494
3-way ANOVA: Max Branch Length	Fig. 10D		Fig. 11D	
Effect of Sex	$F_{1,38}$ = 0.3227	0.5733	$F_{1,39}$ = 1.357	0.2511
Effect of Stress	$F_{1,38}$ = 0.07322	0.7882	$F_{1,39}$ = 2.758e-005	0.9958
Effect of EtOH	$F_{1,38}$ = 1.972	0.1683	$F_{1,39}$ = 0.1962	0.6603
Sex x Stress Interaction	$F_{1,38}$ = 1.748	0.1940	$F_{1,39}$ = 2.093	0.1560
Sex x EtOH Interaction	$F_{1,38}$ = 0.5926	0.4462	$F_{1,39}$ = 0.3419	0.5621
Stress x EtOH Interaction	$F_{1,38}$ = 0.0006834	0.9793	$F_{1,39}$ = 0.06454	0.8008
Sex x Stress x EtOH Interaction	$F_{1,38}$ = 2.216	0.1499	$F_{1,39}$ = 2.995	0.0914
3-way ANOVA: %CD68 Colocalization with Microglia	Fig. 10E		Fig. 11E	
Effect of Sex	$F_{1,40}$ = 0.5531	0.4614	$F_{1,40}$ = 0.7516	0.3911
Effect of Stress	$F_{1,40}$ = 0.06827	0.7952	$F_{1,40}$ = 2.476	0.1235

Effect of EtOH	$F_{1,40}$ = 2.103	0.1548	$F_{1,40}$ = 9.145e-005	0.9924
Sex x Stress Interaction	$F_{1,40}$ = 0.5332	0.4695	$F_{1,40}$ = 1.287	0.2634
Sex x EtOH Interaction	$F_{1,40}$ = 1.052	0.3112	$F_{1,40}$ = 0.1927	0.6631
Stress x EtOH Interaction	$F_{1,40}$ = 0.3070	0.5826	$F_{1,40}$ = 0.4248	0.5183
Sex x Stress x EtOH Interaction	$F_{1,40}$ = 0.5365	0.4682	$F_{1,40}$ = 0.01401	0.9064
3-way ANOVA: $iNos^+ Iba1^+$ / $Arg1^+ Iba1^+$	Fig. 10F		Fig. 11F	
Effect of Sex	$F_{1,40}$ = 0.1617	0.6897	$F_{1,40}$ = 1.581	0.2159
Effect of Stress	$F_{1,40}$ = 0.5161	0.4767	$F_{1,40}$ = 0.3160	0.5772
Effect of EtOH	$F_{1,40}$ = 0.3480	0.5586	$F_{1,40}$ = 2.157	0.1498
Sex x Stress Interaction	$F_{1,40}$ = 1.286	0.2636	$F_{1,40}$ = 0.1133	0.7382
Sex x EtOH Interaction	$F_{1,40}$ = 0.8010	0.3762	$F_{1,40}$ = 3.899	0.0553
Stress x EtOH Interaction	$F_{1,40}$ = 4.784	0.0346*	$F_{1,40}$ = 0.2158	0.6448
Sex x Stress x EtOH Interaction	$F_{1,40}$ = 4.422	0.0418*	$F_{1,40}$ = 0.1562	0.6948
2-way ANOVA: $iNos^+ Iba1^+$ / $Arg1^+ Iba1^+$, Males	Fig. 10F			
Effect of Stress	$F_{1,20}$ = 1.413	0.2485		
Effect of EtOH	$F_{1,20}$ = 0.9079	0.3520		
Stress x EtOH Interaction	$F_{1,20}$ = 7.579	0.0123*		
Šídák's multiple comparisons test: $iNos^+ Iba1^+$ / $Arg1^+ Iba1^+$, Males	Fig. 10F			
No Stress/No EtOH v. No Stress/EtOH	t = 1.273	0.3879		
Stress/No EtOH v. Stress/EtOH	t = 2.620	0.0325*		
No EtOH/No Stress v. No EtOH/Stress	t = 2.787	0.0226*		
EtOH/No Stress v. EtOH/Stress	t = 1.106	0.4842		
2-way ANOVA: $iNos^+ Iba1^+$ / $Arg1^+ Iba1^+$, Females	Fig. 10F			
Effect of Stress	$F_{1,20}$ = 1.099	0.7438		
Effect of EtOH	$F_{1,20}$ = 0.05922	0.8102		
Stress x EtOH Interaction	$F_{1,20}$ = 0.004514	0.9471		

3-way ANOVA: %iNos Colocalization with Iba1	Fig. 10G		Fig. 11G	
Effect of Sex	$F_{1,40}$ = 0.5712	0.4542	$F_{1,40}$ = 0.04056	0.8414
Effect of Stress	$F_{1,40}$ = 0.2170	0.6439	$F_{1,40}$ = 1.918	0.1738
Effect of EtOH	$F_{1,40}$ = 7.384	0.0097**	$F_{1,40}$ = 1.601	0.2130
Sex x Stress Interaction	$F_{1,40}$ = 0.7314	0.3975	$F_{1,40}$ = 0.5587	0.4591
Sex x EtOH Interaction	$F_{1,40}$ = 2.584	0.1158	$F_{1,40}$ = 0.1108	0.7410
Stress x EtOH Interaction	$F_{1,40}$ = 0.5941	0.4454	$F_{1,40}$ = 0.000000249	0.9996
Sex x Stress x EtOH Interaction	$F_{1,40}$ = 1.262	0.2680	$F_{1,40}$ = 0.5626	0.4576
3-way ANOVA: %Arg1 Colocalization with Iba1	Fig. 10H		Fig. 11H	
Effect of Sex	$F_{1,40}$ = 0.01904	0.8909	$F_{1,40}$ = 0.0002887	0.9865
Effect of Stress	$F_{1,40}$ = 0.002881	0.9575	$F_{1,40}$ = 0.6238	0.4343
Effect of EtOH	$F_{1,40}$ = 1.394	0.2446	$F_{1,40}$ = 5.828	0.0204*
Sex x Stress Interaction	$F_{1,40}$ = 2.870	0.0980	$F_{1,40}$ = 0.1274	0.7231
Sex x EtOH Interaction	$F_{1,40}$ = 2.330	0.1347	$F_{1,40}$ = 1.216	0.2767
Stress x EtOH Interaction	$F_{1,40}$ = 0.5758	0.4524	$F_{1,40}$ = 0.2534	0.6175
Sex x Stress x EtOH Interaction	$F_{1,40}$ = 1.017	0.3193	$F_{1,40}$ = 0.007520	0.9313
